# Antitumour effects of SFX-01 molecule in combination with ionizing radiation in preclinical and in vivo models of rhabdomyosarcoma

**DOI:** 10.1186/s12885-024-12536-8

**Published:** 2024-07-08

**Authors:** Simona Camero, Luisa Milazzo, Francesca Vulcano, Federica Ceccarelli, Paola Pontecorvi, Francesca Pedini, Alessandra Rossetti, Elena Sofia Scialis, Giulia Gerini, Fabrizio Cece, Silvia Pomella, Matteo Cassandri, Antonella Porrazzo, Enrico Romano, Claudio Festuccia, Giovanni Luca Gravina, Simona Ceccarelli, Rossella Rota, Lavinia Vittoria Lotti, Fabio Midulla, Antonio Angeloni, Cinzia Marchese, Francesco Marampon, Francesca Megiorni

**Affiliations:** 1 Department of Experimental Medicine, “Sapienza” University of Rome, Rome, Italy; 2 Department of Oncology and Molecular Medicine, Italian National Institute of Health (ISS), Rome, Italy; 3 Department of Biotechnological and Applied Clinical Sciences, University of L’Aquila, L’Aquila, Italy; 4 Department of Innovative Technologies in Medicine and Dentistry, University “G. D’Annunzio” Chieti - Pescara, Chieti, Italy; 5 Department of Clinical Sciences and Translational Medicine, University of Rome Tor Vergata, Rome, Italy; 6 Department of Oncohematology, Bambino Gesù Children’s Hospital, IRCCS, Rome, Italy; 7 Department of Radiological, Oncological and Pathological Sciences, “Sapienza” University of Rome, Rome, Italy; 8 Department of Sense Organs, “Sapienza” University of Rome, Rome, Italy; 9 Department of Maternal Infantile and Urological Sciences, “Sapienza” University of Rome, Rome, Italy

**Keywords:** Rhabdomyosarcoma, SFX-01, Sulforaphane, Radiotherapy, Cell cycle arrest, Oxidative stress, 3D tumorspheres

## Abstract

**Background:**

Despite a multimodal approach including surgery, chemo- and radiotherapy, the 5-year event-free survival rate for rhabdomyosarcoma (RMS), the most common soft tissue sarcoma in childhood, remains very poor for metastatic patients, mainly due to the selection and proliferation of tumour cells driving resistance mechanisms. Personalised medicine-based protocols using new drugs or targeted therapies in combination with conventional treatments have the potential to enhance the therapeutic effects, while minimizing damage to healthy tissues in a wide range of human malignancies, with several clinical trials being started. In this study, we analysed, for the first time, the antitumour activity of SFX-01, a complex of synthetic d, l-sulforaphane stabilised in alpha-cyclodextrin (Evgen Pharma plc, UK), used as single agent and in combination with irradiation, in four preclinical models of alveolar and embryonal RMS. Indeed, SFX-01 has shown promise in preclinical studies for its ability to modulate cellular pathways involved in inflammation and oxidative stress that are essential to be controlled in cancer treatment.

**Methods:**

RH30, RH4 (alveolar RMS), RD and JR1 (embryonal RMS) cell lines as well as mouse xenograft models of RMS were used to evaluate the biological and molecular effects induced by SFX-01 treatment. Flow cytometry and the modulation of key markers analysed by q-PCR and Western blot were used to assess cell proliferation, apoptosis, autophagy and production of intracellular reactive oxygen species (ROS) in RMS cells exposed to SFX-01. The ability to migrate and invade was also investigated with specific assays. The possible synergistic effects between SFX-01 and ionising radiation (IR) was studied in both the in vitro and in vivo studies. Student’s t-test or two-way ANOVA were used to test the statistical significance of two or more comparisons, respectively.

**Results:**

SFX-01 treatment exhibited cytostatic and cytotoxic effects, mediated by G2 cell cycle arrest, apoptosis induction and suppression of autophagy. Moreover, SFX-01 was able to inhibit the formation and the proliferation of 3D tumorspheres as monotherapy and in combination with IR. Finally, SFX-01, when orally administered as single agent, displayed a pattern of efficacy at reducing the growth of tumour masses in RMS xenograft mouse models; when combined with a radiotherapy regime, it was observed to act synergistically, resulting in a more positive outcome than would be expected by adding each exposure alone.

**Conclusions:**

In summary, our results provide evidence for the antitumour properties of SFX-01 in preclinical models of RMS tumours, both as a standalone treatment and in combination with irradiation. These forthcoming findings are crucial for deeper investigations of SFX-01 molecular mechanisms against RMS and for setting up clinical trials in RMS patients in order to use the SFX-01/IR co-treatment as a promising therapeutic approach, particularly in the clinical management of aggressive RMS disease.

**Supplementary Information:**

The online version contains supplementary material available at 10.1186/s12885-024-12536-8.

## Background

Rhabdomyosarcoma (RMS) is the most common soft tissue sarcoma in the paediatric population, representing about 50% of all sarcomas and 5% of cancer pathology in children [1, 2]. The two main subtypes of this high-grade, malignant solid tumour are alveolar RMS (ARMS) and embryonal RMS (ERMS) [3]. ARMSs are more aggressive than ERMSs and are characterised by recurrent translocations, t(2;13)(q35;q14) or t(1;13)(p36;q14) that juxtapose PAX3 (chromosomes 2) or PAX7 (chromosomes 1) genes with FOXO1 gene (chromosome 13), so generating PAX3/PAX7-FOXO1 fusion proteins [4, 5]. ERMSs, the most common subtype, typically show aneuploidy, loss of heterozygosity of the short arms of chromosome 11 (11p15) and/or mutations in the RAS pathway components [3, 6]. Multimodal therapy, including surgery, chemotherapy (vincristine, actinomycin D, and ifosfamide or cyclophosphamide) and radiotherapy, is the standard treatment of RMS tumours [7–9]. In general, the 5-year event-free survival (EFS) rates for localized RMS range from approximately 60–80%, depending on the specific characteristics of the tumour and the effectiveness of treatment. For metastatic RMS, the 5-year EFS rates tend to be lower compared to localized RMS, often ranging from around 20–50%, depending on the extent of metastasis and response to treatment [10–13]. Chemo/radiotherapy frequently fail due to the selection and proliferation of resistant tumour cells [14–16], therefore, innovative therapeutic protocols are urgently required to enhance the efficacy of conventional therapies and improve patient prognosis.

Recently, the therapeutic activity of sulforaphane (SFN), an isothiocyanate isolated from cruciferous vegetables, such as broccoli, cabbage, and cauliflower, has been highlighted in clinical context [17]. Several studies have demonstrated SFN efficacy for its anti-inflammatory, antidiabetic, antioxidant, cardioprotective and hepatoprotective activity [18–22], so helping to protect cells from oxidative stress and inflammation. The anticancer properties of SFN have also been investigated in a variety of solid tumours, including breast [23, 24], ovarian [25], lung [26], prostate [27], gastric [28, 29] and oral squamous cell carcinomas [30]. The ability of SFN to reduce cell proliferation and trigger apoptosis has also been described in ARMS cellular models [31]. More recently, SFX-01, a complex of synthetic d, l-sulforaphane stabilised in alpha-cyclodextrin (Evgen pharma plc, UK), has been developed for pharmaceutical purposes. SFX-01 has been showed to have antitumour properties in preclinical models of breast cancer and glioblastoma [32–34]. In particular, Simões et al. demonstrated that SFX-01 acts against resistant breast stem-like cells in both patient samples and patient-derived xenograft tumours [32]. A phase II clinical trial (NCT02970682) also evidenced the safety, tolerability and efficacy of SFX-01 in patients with ER + HER2- metastatic breast cancer and its antitumour activity in combination with endocrine therapies [33]. Colapietro and colleagues established that SFX-01 reduced the survival and stemness of glioblastoma cells, induced apoptosis and impaired in vivo tumour growth [34].

In the present study we analysed the biological and molecular effects of SFX-01 in RH30, RH4, RD and JR1 cells, in vitro models of ARMS and ERMS, by also evaluating the possible synergistic effects between SFX-01 and ionising radiation (IR). Moreover, we tested the efficacy of the simultaneous SFX-01/IR treatment on 3D tumour spheroids derived from parental and clinically relevant radioresistant RMS cell lines. Finally, we assessed the in vivo antitumour activity of SFX-01 as single agent and in combination with radiotherapy in a mouse xenograft model of RMS.

## Methods

### Cell cultures

Human in vitro models of ARMS (RH30 and RH4) and ERMS (RD and JR1) cell lines used in this study were maintained in High Glucose Dulbecco’s Modified Eagle Medium (DMEM-HG) (Sigma-Aldrich, St. Louis, MO, USA) supplemented with 10% Foetal bovine serum (FBS) (Corning, New York, NY, USA), 2 mM L-glutamine (Corning), 100 IU/ml penicillin and 100 µg/ml streptomycin (Corning). Specifically, RH30 and RD cells were purchased from American Type Culture Collection, whilst RH4 and JR1 cells were kindly provided by Dr. Rota R (Bambino Gesù Children’s Hospital, Rome, Italy). Human foetal myoblast (HFM) cells (kindly provided in 2013 by Dr Felsani A.) were cultured in DMEM-HG supplemented with 20% FBS. Clinically relevant radioresistant (RR) RMS cells RH30 RR and RD RR, previously obtained by our group [35] were also maintained in complete DMEM-HG. All cell lines were grown at 37 °C in 5% CO_2_.

### Reagents

SFX-01, a complex of synthetic d, l-sulforaphane stabilized in alpha-cyclodextrin (molecular weight: Sulforaphane: 177.29 g/mole, Alpha-cyclodextrin: 972.86 g/mole), was provided from Evgen Pharma plc. (Nether Alderley, UK) as lyophilized powder and reconstituted in dimethyl sulfoxide (DMSO) (Santa Cruz Biotechnology, Dallas, TX, USA) to a final concentration of 10 mM (equivalent to 1.54 mM Sulforaphane). DMSO alone was used as negative control in untreated cells at 0.1% (v/v) concentration.

### Viability and proliferation assays

Viability of RH30, RD, RH4 and JR1 cells was measured using the MTT assay. Specifically, cells were seeded as sextuplicate into 96-well plates and treated with different concentrations of SFX-01 (from 0.125 to 20 µM) or DMSO at the maximum amount used for SFX-01 delivery. After 72 h of SFX-01 exposure, 0.5 mg/ml MTT solution (Sigma-Aldrich) was added to each well for 3 h (h). Blank cell-free control was also included. Then, plates were incubated at 37 °C for 10 min (min) with 100 µl of DMSO. Absorbance was measured at 550 nm, with reference at 630 nm, using a microtiter plate reader (Select Science). Results were plotted as mean ± SD of cell viability percentage vs. logarithm of the concentration of two independent experiments.

Cell proliferation of RH30, RD, RH4, JR1 and HFM cells was evaluated with trypan blue dye exclusion method [36].

### Morphological assessment by Giemsa assay

Morphological changes mediated by SFX-01 treatment on RH30, RD, RH4, JR1 and HFM morphology were evaluated by the standard Giemsa staining. Briefly, after being cultured with SFX-01 or DMSO for 48 h, cells were fixed by cold methanol for 15 min and stained in 10% Giemsa solution (Sigma-Aldrich) for 15 min at room temperature (RT). After washing in tap water, cells were allowed to air dry and photographed with EVOS XL Core Imaging System (Thermo Fisher Scientific, Waltham, MA, USA) at 4x,10x or 20x magnification.

### Cell cycle, apoptosis and ROS production analysis by flow cytometry

For cell cycle analysis, a BD Cycletest Plus DNA Kit (BD Biosciences, San Jose, CA) was used for DNA staining. Following trypsinization, cells were resuspended to a final concentration of 1 × 10^6^ cells/ml and treated with reagent kit, according to the manufacturer’s instructions. Samples were stained with propidium iodide (PI) solution, and the cell cycle status was analysed by collecting at least 50,000 events for each sample by using a BD FACSCalibur flow cytometer (BD Biosciences). Cell cycle distribution was analysed using the Mod-Fit LT 3.0 software (Verity Software House, Topsham, ME, USA).

Apoptosis was analysed by using PE Annexin V Apoptosis Detection Kit I (BD Biosciences), following the manufacturer’s instructions. Floating and attached cells were collected, washed twice in cold 1x Annexin V Binding Buffer and suspended in 1x Annexin V Binding Buffer at 2 × 10^6^ cells/ml. Approximately 2 × 10^5^ cells were stained with Annexin V and 7-Amino-Actinomycin (7-AAD) for 15 min at RT in the dark. Fluorescence intensities of Annexin V and 7-AAD of mocked control or treated cells were analysed using a BD FACSCalibur (BD Biosciences). Data were analysed using Cell Quest Pro software (BD Biosciences).

For reactive oxygen species (ROS) detection, Total ROS Assay Kit 520 nm (Invitrogen) was used according to the manufacturer’s instructions. Briefly, 5 × 10^5^ RH30 and RD cells were seeded in 25 cm^2^ flasks and treated with SFX-01 or DMSO for 48 h. Harvested cells were resuspended in ROS assay dye solution and incubated for 1 h at 37 °C in 5% CO_2_ incubator. At least 5 × 10^3^ events for each sample were analysed by using a BD FACSCalibur flow cytometer (BD Biosciences). Median fluorescence intensity, used to compare total ROS produced by SFX-01-treated compared to negative control cells, was analysed using Cell Quest software (BD Biosciences).

### Transient transfection of small interfering RNA and SFX-01 treatment

RH30 and RD cells were seeded at 1.3 × 10^5^ cells/well in 12-well plates; small interfering RNA (siRNA) against human cyclin D1 or siRNA negative control (si-cyclin D1, sc-44257; si-NC, sc-37007 by Santa Cruz Biotechnology) were combined with RNAiMAX (Invitrogen) at 60 nM final concentration following the manufacturer’s protocol. si-cyclin D1 is a pool of 3 target-specific 19–25 nt siRNAs designed to specifically knock down gene expression. Twenty-four h after the transient transfection RH30 and RD cells were treated with SFX-01 or DMSO. After additional 48 h RMS cells were collected for the subsequent assays.

### RNA extraction and quantitative real time PCR

Total RNA was isolated from RH30 and RD cell lines treated or not with SFX-01 by using TRIsure™ (BIOLINE, London, UK) according to the manufacturer’s instructions. One microgram of total RNA was reverse transcribed using the SensiFAST™ cDNA Synthesis Kit (BIOLINE) and analysed by using quantitative Real Time PCR (q-PCR) on a StepOne Real Time System (Applied Biosystems, Foster City, CA, USA) machine. Transcript levels of p21, NRF2, SOD2, CAT, GPx4 and GST-M1 were analysed using SensiFAST™ SYBR Hi-ROX Kit (BIOLINE). Cyclin D1 gene expression was quantified by using a specific TaqMan Real-Time Gene Expression Assay (Applied Biosystems). GAPDH levels were used to normalise the results. Primer sequences are available upon request. Comparative Ct method was used to calculate the relative gene expression. Results were expressed as fold change compared to negative control cells. Each sample was run in triplicate, in at least three independent experiments.

### Total, nuclear and cytoplasmic protein extraction and Western blotting

RH30, RD, RH4 and JR1 cells were collected at different time after specific treatment and were lysed as previously described [37]. For nuclear and cytoplasmic protein extraction, RH30 and RD cells exposed for 72 h to SFX-01 or DMSO were processed as previously described [38]. For Western blotting, 30–80 µg of protein were separated on 8–15% sodium dodecyl sulphate-polyacrylamide gel (SDS-PAGE) and transferred onto polyvinylidene fluoride (PVDF) membranes (Amersham, Chicago, IL, USA). Filters were blocked with 5% non-fat dry Milk or 3% bovine serum albumin (BSA) in phosphate buffered saline with Tween 20 (PBS-T) for at least 30 min at RT and incubated over-night at + 4 °C or 2 h at RT with the following primary antibodies: cyclin B1, cyclin D1, p16, p21, p27 (Santa Cruz Biotechnology), cleaved PARP, γ-H2AX (Cell Signalling Technology, Danvers, MA), p62 (BD Biosciences) and LC3I/II (Sigma). Antibody against tubulin (Santa Cruz Biotechnology) was used as a loading control whilst Lamin B1 (Abcam, Cambridge, UK) and β-actin (Sigma) were used as control for nuclear and cytoplasmic fractions respectively. The different filters were cut to have a more precise hybridization and not to waste precious primary antibodies. Images were acquired by ChemiDoc XRS+ (Bio-Rad, Hercules, CA, USA).

### Protein simple WES western analysis

Specific high molecular weight proteins were analysed by using Protein Simple WES Western technology as previously reported [39]. Briefly, 500 ng of protein simple was mixed with 5x fluorescent master mix (Protein Simple/Bio-Techne, Minneapolis, MN, USA) to achieve a final concentration of 1x master mix buffer, according to manufacturer’s instructions. Samples were then denatured at 95 °C for 5 min. Antibody diluent, protein normalising reagent, primary and secondary antibodies, chemiluminescent substrates, 3 µl of sample, and 500 µl of wash buffer were prepared and dispensed into the assay plate. Assay plates were loaded into the instrument and proteins were separated within individual capillaries. Protein detection and digital images were collected and analysed with Compass software (Protein Simple) and data were reported as area under the peak, which represents the intensity of the signal. The primary antibodies phospho-ATM, phospho-DNA-PK_cs_, DNA-PK_cs_ (Cell Signaling Technology), and ATM (Santa Cruz Biotechnology) were mixed with vinculin (Sigma-Aldrich). Appropriate anti-mouse HRP and anti-rabbit HRP secondary antibodies from Protein Simple were used.

### Migration, invasion and wound-healing assays

For the trans-well cell migration/invasion assay, RH30 and RD cells pre-treated or not with SFX-01 for 48 h were plated in serum-free DMEM-HG at 5 × 10^4^ cell/well in the upper compartment of the BD FalconTM Cell Culture Inserts with 8 μm pore polycarbonate filters (Falcon, New York, NY, USA) placed into a 24-well culture plate. The lower compartment contained DMEM-HG with 10% FBS that was used as chemoattractant. For invasion assay, filters were coated with Matrigel. After over-night incubation at 37 °C migrated cells were fixed in 100% methanol and stained with 0.1% Crystal violet dye. Non-migrating cells on the upper surface of the membrane were removed with cotton swabs. Five random fields were photographed with EVOS XL Core Imaging System (Thermo Fisher Scientific) at 10x magnification, and the number of cells was calculated by using ImageJ software (1.54d version). Data were plotted as mean ± SD of migrated cells of three independent experiments each performed in duplicate.

To evaluate the spontaneous cell migration, wound-healing assay was also performed. RH30 and RD cells were plated at 2.5 × 10^5^ cell/well in 12-well plates. After 24 h the scratch was performed in each well and RMS cells were treated with or without SFX-01. Images were collected every 3 h by using IncuCyte^®^ S3 (Sartorius, Goettingen, Germany) until the wound in the negative controls was covered and repaired. Wound closure was calculated with the ImageJ software. Statistical analysis on the closure of the wound area was performed considering two independent experiments.

### 3D tumour sphere formation

To obtain three dimensional (3D) rhabdospheres, cells were grown in ultra-low attachment plates (Corning) as previously described [40]. Two different experiments were performed to assess both the cytostatic and cytotoxic effect induced by SFX-01 on 3D rhabdospheres. Specifically, SFX-01 or DMSO were added to the medium after 24 h prior the sphere formation or we allowed the spheres to form and then treated them with increasing concentrations of SFX-01. Cells were photographed at 10x magnification at 7 and 14 days and after being disrupted in a single cell suspension were counted by using trypan blue. To assess the effect of SFX-01/IR combined treatment on 3D rhabdosphere formation, RH30, RD, RH30 RR and RD RR were pre-treated with SFX-01 or DMSO for 24 h and then were irradiated or not with a single dose of 4 Gy. After 14 days cells were photographed under EVOS XL Core Imaging System at 10x magnification. Different morphological parameters, including diameter, area and volume of 3D spheroids, were analysed by using AnaSP [41], an open-source image-based software tool, freely available, specifically developed for automatic computation of specific features of multicellular 3D spheroids. Briefly, data were extracted after segmenting the acquired images to obtain a binary mask of each spheroid.

### Electron microscopy

To evaluate the presence of autophagosomes, autolysosomes or other vacuolar structures, we performed transmission electron microscopy (TEM) experiments, as previously described [42]. Briefly, RH30 and RD cells treated with SFX-01 (10–20 µM) or DMSO for 72 h were fixed in 2% glutaraldehyde (Electron Microscopy Sciences, Hatfield, PA, USA) in 0.1 M PBS for 24 h at + 4 °C. After collection, cells were washed three times in PBS, post-fixed for 2 h in 1% OsO_4_ (Electron Microscopy Sciences), dehydrated in graded acetone solution and embedded in Epon-812 epoxy resin (Electron Microscopy Sciences) following standard procedures. Ultrathin Sect. (60 nm) were cut with a Reichert ultramicrotome (Leica Microsystems, Wetzlar, Germany), mounted on copper grids, counterstained with uranyl-acetate replacement stain and lead citrate (Electron Microscopy Sciences) and finally examined with a Fei-Philips Morgagni 268D transmission electron microscope (FEI, Eindhoven, The Netherlands).

### Irradiation

RH30, RD, RH4 and JR1 cells were seeded in 25 cm^2^ flasks, cultured for 24 h with SFX-01 and irradiated using a Gammacell Exactor 40 (Nordion) equipped with two Cs-137 sources at a total single dose of 4 Gy. At 48 h post SFX-01 treatment and 24 h after radiation exposure, cells were collected for the subsequent analysis.

### Clonogenic assay

For colony formation assay, RH30, RD, RH4 and JR1 cells were seeded in 6-well plates in triplicate at low density. Drug-free medium was replaced every three days and after 12 days colonies were fixed in ice-cold methanol and stained with 0.1% crystal violet solution in 25% methanol. After air drying over-night, colonies were photographed by using the ChemiDoc™ XRS+ (Bio-rad). For the quantitative analysis, crystal violet was solubilised in 30% acetic acid in water for 15 min at RT and the absorbance was measured by using the Biochrom Libra S22 UV/VIS spectrophotometer (Biochrom, Berlin, Germany) at wavelength of 595 nm; 30% acetic acid in water was used as the blank. The results were plotted as mean of OD_treatment_ / OD_control_ ± SD of at least two independent experiments each performed in triplicate.

### Modified high density survival assay (MHDSA)

To further assess the cell survival after SFX-01 treatment and irradiation we used and adapted the modified high density survival assay (MHDSA) by Kuwahara [43]. Briefly, RH30 and RD cells were seeded (5 × 10^5^) in 25 cm^2^ flasks, cultured for 24 h with 10 µM SFX-01 and exposed to a single dose of 4 Gy. Twenty-four hours after irradiation 1/10 of cells of each flask were seeded in a new 25 cm^2^ flasks and incubated for 5 days. This step was repeated twice and at the end of incubation cells were collected and counted by trypan blue dye exclusion test.

### In vivo xenograft experiments

The recommendations of the European Community (EC) guidelines (2010/63/UE and DL 26/2014 for the use of laboratory animals) and the Italian National Institute of Health guidelines, complying with the Italian Government Regulation n.116 27 January 1992 for the use of laboratory animals were followed to perform in vivo experiments. The in vivo study was approved by the Ethics committee of the Italian National Institute of Health with the code 221/2022-PR (D9997.140) on 4 April 2022. All the experiments on mice were performed at the Italian National Institute of Health (ISS), Rome, Italy. Before any invasive manipulation, mice were anaesthetised with a mixture of 25 mg/ml ketamine/5 mg/ml xylazine. Exponentially growing RH30 (3 × 10^6^/200 µl) or RD cells (10 × 10^6^/200 µl) were resuspended in saline solution and subcutaneously injected in the leg of 45-day-old female nude CD1 mice (supplied by the Envigo RMS S.r.l, San Pietro al Natisone, Udine, Italy), by using a 21-gauge needle on a tuberculin syringe. Treatments started when tumours reached a volume of 0.2 cm^3^. Before tumour inoculation, mice were randomly assigned to four experimental groups, each consisting of three mice. The control group received 200 µl of carrier solution by mouth, the second group received SFX-01 (50 mg/kg, equivalent to 7.7 mg/kg Sulforaphane) by mouth once daily for five consecutive days, the third group of mice were irradiated at room temperature every other day with a single dose of 2 Gy using the Gammacell Exactor 40 (Nordion) equipped with two Cs-137 sources and the last group received SFX-01 and IR. Prior to irradiation, mice were anaesthetised and protected from off-target radiation by a 3 mm lead shield. During treatment, mice with significant body weight loss approaching (10–15%) were euthanized early per protocol, by using Carbon Dioxide, following the AVMA Guidelines for the Euthanasia of Animals (AVMA, 2019). The effects of treatments on tumour growth were evaluated as follows: tumour volume, measured during and at the end of the experiment with a Vernier calliper (length x width), was expressed in cm^3^ according to the formula length x (width)^2^ / 2; tumour weight was assessed only at the end of the experiment.

### Statistical analysis

Statistical analyses were performed by using unpaired Student’s t-test or two-way ANOVA. Tukey’s correction was used for multiple comparison. A probability (p) ≤ 0.05 was considered statistically significant. All the results are presented as means ± SD of at least three independent experiments unless otherwise stated.

## Results

### SFX-01 treatment drastically reduces the proliferation of RMS cells

**Fig. 1:**
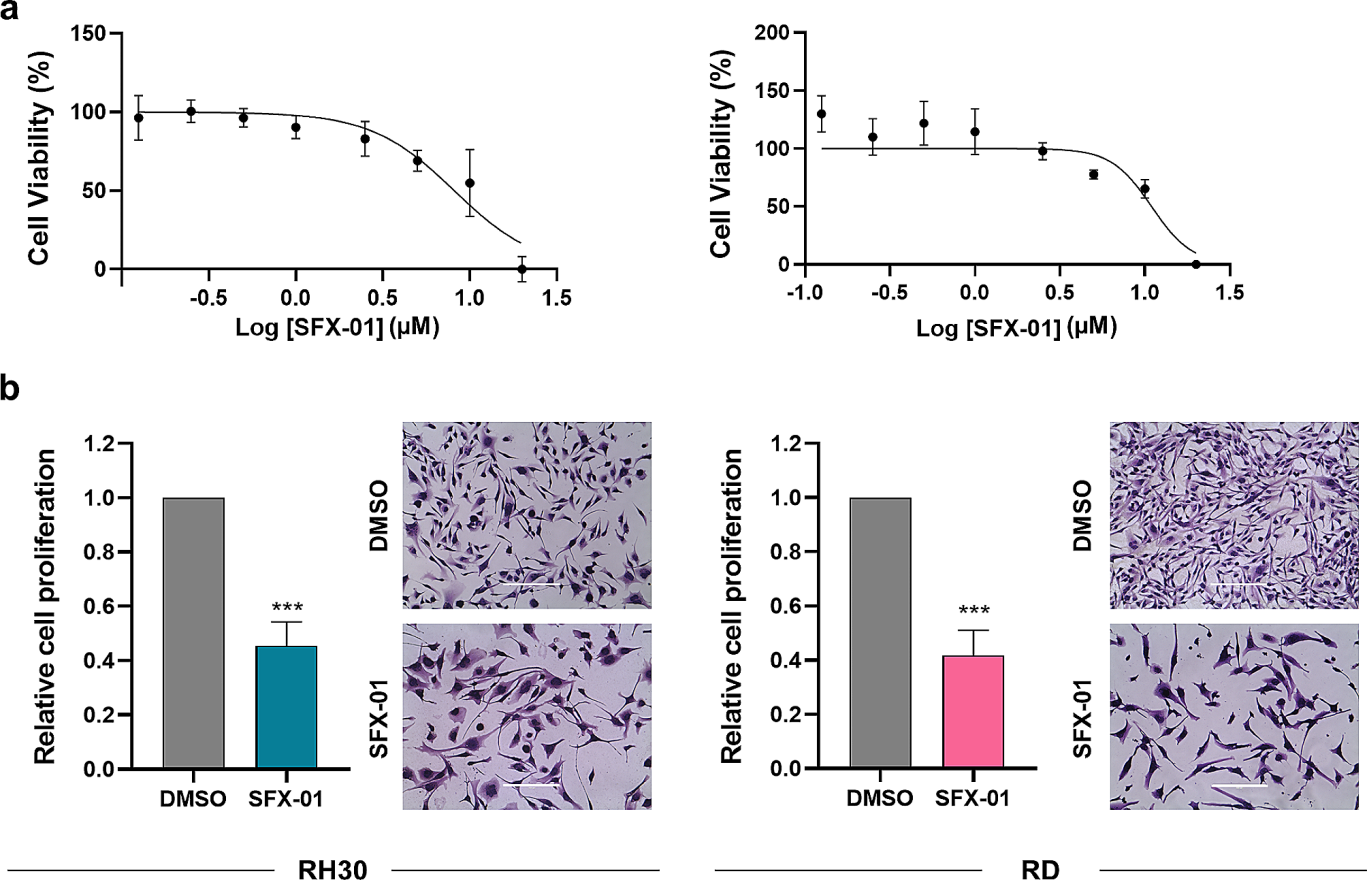
Proliferation and morphology evaluation in RMS cells exposed to SFX-01. **(a)** MTT assay performed on RH30 and RD cells treated for 72 h with increasing concentrations of SFX-01. Each point is the mean ± SD of two independent experiments each performed in sextuplicate. **(b)** Trypan blue assay showing RH30 and RD cell proliferation at 72 h post SFX-01 exposure (10 µM) expressed as fold increase over DMSO treated cells, arbitrarily set at 1. Histograms represent mean values ± SD of at least three independent experiments. Statistical analyses were performed by using Student’s t-test: ***, *p* < 0.001 vs. DMSO. Images showing the morphological assessment by Giemsa staining. Scale bar: 200 μm

To assess the antitumour effect of SFX-01 in two RMS in vitro models, we first performed the MTT assay treating RH30 and RD cells with increasing concentration of SFX-01 (0.125, 0.25, 0.5, 1, 2.5, 5, 10 and 20 µM) or DMSO, used as negative control, for 72 h. As shown in Fig. 1a, the metabolic activity, which directly correlates with cell viability and proliferation, was inhibited in both the cell lines in a dose dependent manner, and for the subsequent experiments we used the concentration of 10 µM, corresponding to the IC_50_ value. In addition, the strong reduction of cell proliferation was confirmed by Trypan blue dye exclusion assay and morphological assessment by Giemsa staining Indeed, RH30 and RD cells exposed to 10 µM SFX-01 for 72 h presented a growth inhibition rate of about 60%, clearly shown also by the light microscope images, and moreover, both the cell lines treated with SFX-01 exhibited cellular bodies much larger than the mocked control cells. Similar results were obtained in two additional in vitro models of RMS tumours: the RH4 cell line, as a model of ARMS, and the JR1 cell line, as a model of ERMS (Supplementary Fig. 1a-b). Moreover, in accordance with the enlarged cell morphology, the myogenic regulatory factors MyoD and Myogenin did not increase their levels after SFX-01 treatment (data not shown).

**Fig. 2:**
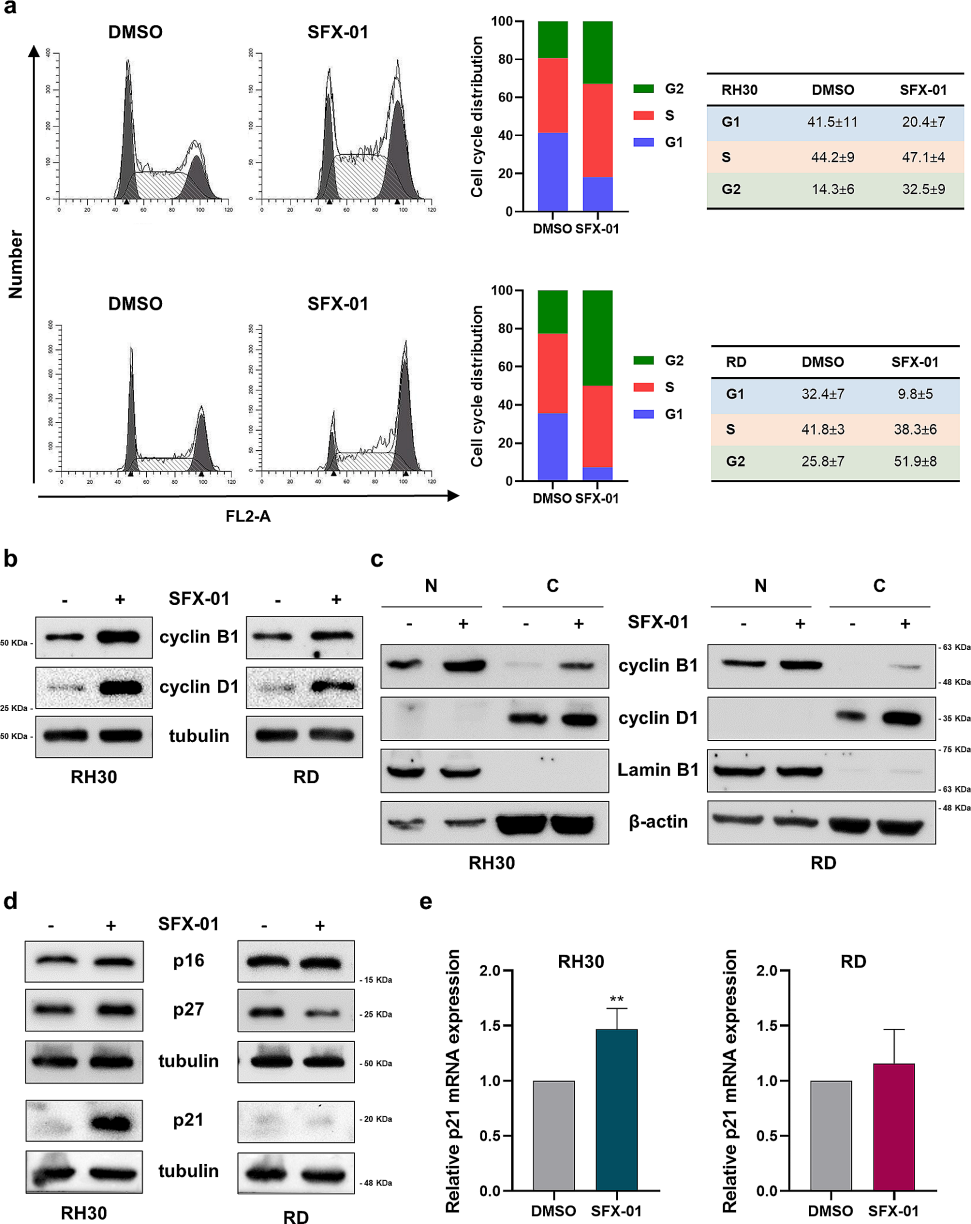
Analysis of cell cycle progression in SFX-01 treated RH30 and RD cells. **(a)** Cell cycle distribution of RH30 (upper panels) and RD (lower panels) cells treated or not for 72 h with 10 µM SFX-01. Representative plots of FACS analysis; histograms showing the mean values of four independent experiments; tables reporting the mean values ± SD for each cell cycle phase. **(b)** Western blot assay of cyclin B1 and cyclin D1 in RMS cells exposed to 10 µM SFX-01 or DMSO. Tubulin expression was used as loading control. Representative blots of three independent experiments. Western blots were cropped to improve the conciseness of the results. The original Western blot images can be found in Supplementary File_uncropped. **(c)** Western blot analysis performed on nuclear (N) and cytoplasmic (C) protein extracts from RH30 and RD cells treated for 72 h with SFX-01. Lamin B1 was used as control for nuclear fraction, whilst β-actin for cytoplasmic control. Representative blots of two independent experiments. Western blots were cropped to improve the conciseness of the results. The original Western blot images can be found in Supplementary File_uncropped. **(d)** Western blot of several cell cycle inhibitors. Tubulin expression was used as loading control. Representative blots of three independent experiments. Western blots were cropped to improve the conciseness of the results. The original Western blot images can be found in Supplementary File_uncropped. **(e)** q-PCR analysis showing p21 mRNA levels in RH30 and RD cells exposed to SFX-01 expressed as fold increase over DMSO treated cells, arbitrarily set at 1. Transcript levels were normalised to GAPDH mRNA. Results are expressed as mean values ± SD of four independent experiments, each performed in triplicate. Statistical analyses were performed by using Student’s t-test: **, *p* < 0.01 vs. DMSO

To evaluate the specificity of SFX-01 on RMS cells, we also treated HFM for 72 h and we demonstrated that SFX-01 affected neither the proliferation nor the morphology of these primary cells (Supplementary Fig. 2). To better understand the inhibition of cell proliferation, we investigated the cell cycle distribution in RMS cells treated for 72 h with SFX-01 or DMSO. As suggested by the morphological changes highlighted by Giemsa assay, flow cytometry analysis showed that SFX-01 induced a significant G2 cell cycle arrest (RH30 *p* = 0.004; RD *p* < 0.001) and the concomitant decrease of cell percentage in G1 phase (RH30 *p* < 0.001; RD *p* < 0.001) in both the cell lines (Fig. 2a). We also analysed the protein levels of several cell cycle markers by Western blotting (Fig. 2b). In agreement with the G2 arrest detected by the cytofluorimetric analysis, we observed the up-regulation of cyclin B1 expression in RMS cells exposed to SFX-01 compared to DMSO. Surprisingly, cyclin D1 levels also increased after SFX-01 treatment, however the analysis of the nuclear and cytoplasmic protein fraction revealed that cyclin D1 was localised only in the cytosol of RH30 and RD cells, whilst cyclin B1 was mainly expressed in the nuclear compartment (Fig. 2c). Upregulation of both cyclin B1 and cyclin D1 protein levels was also confirmed in RH4 and JR1 cells exposed to SFX-01 (Supplementary Fig. 1c).

To better understand the specific function of cyclin D1 in SFX-01-mediated effects in RMS we silenced cyclin D1 in SFX-01-exposed cells. As reported in Supplementary Fig. 3, cyclin D1 depletion and SFX-01 treatment promoted significant reduction of cell number compared to mocked controls or single treatment in RH30 cells, suggesting that cyclin D1 may counteract the cytostatic effect induced by SFX-01 in alveolar RMS (Supplementary Fig. 3b). Knocking-down cyclin D1 did not appear to alter the inhibition of proliferation mediated by SFX-01 in RD cells (Supplementary Fig. 3d). Finally, we analysed the expression of several cell cycle inhibitors, which exhibited increased amounts of p16, p27 and p21, at mRNA and/or protein levels, only in RH30 cells (Fig. 2d-e). In RH4 and JR1 cells treated with SFX-01, the expression of the p21 cell cycle master regulator showed a similar tumour subtype-related pattern (Supplementary Fig. 1c).

These data indicate that SFX-01 exerts an antitumour activity in RMS models by reducing the number of actively proliferating cells.

### SFX-01 induces the apoptosis of RMS cells by suppressing autophagy at late stage and increasing oxidative stress

**Fig. 3:**
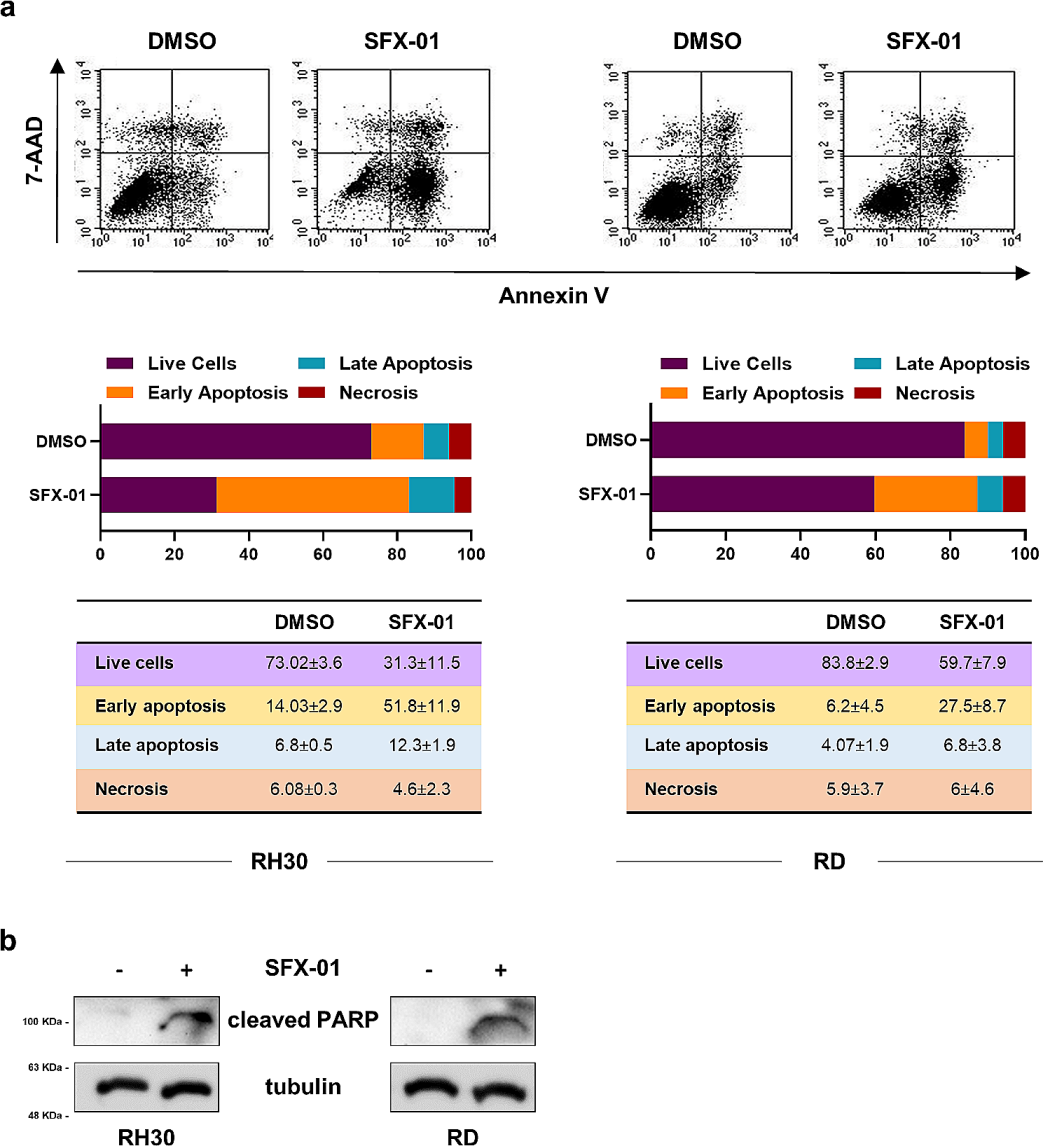
Evaluation of SFX-01 effects on apoptosis in RH30 and RD cells. **(a)** Cytofluorimetric analysis of apoptotic process in RH30 (left panels) and RD (right panels) cells exposed to SFX-01 (10 µM) or DMSO. Plots representing a single experiment; histograms showing the mean values of three independent experiments; tables reporting the mean values ± SD of the different cell populations. **(b)** Relative expression of cleaved PARP protein shown by Western blot assay. Tubulin expression was used as loading control. Representative blots of two independent experiments. Western blots were cropped to improve the conciseness of the results. The original Western blot images can be found in Supplementary File_uncropped

To better understand the antitumour effects mediated by SFX-01 in RMS models, we investigated the apoptotic process after 72 h of treatment. As showed in Fig. 3a, the specific staining with Annexin V and 7-AAD demonstrated that SFX-01 exposure significantly increases the cell percentage in early apoptosis in both RH30 (*p* = 0.001) and RD (*p* < 0.001) compared to negative control cells. Next, we evaluated the modulation of specific apoptosis markers and the immunoblots revealed the slight increase of cleaved PARP (Fig. 3b), even if neither caspase 3 activation nor BCL-2 inhibition were evident (data not shown).

**Fig. 4:**
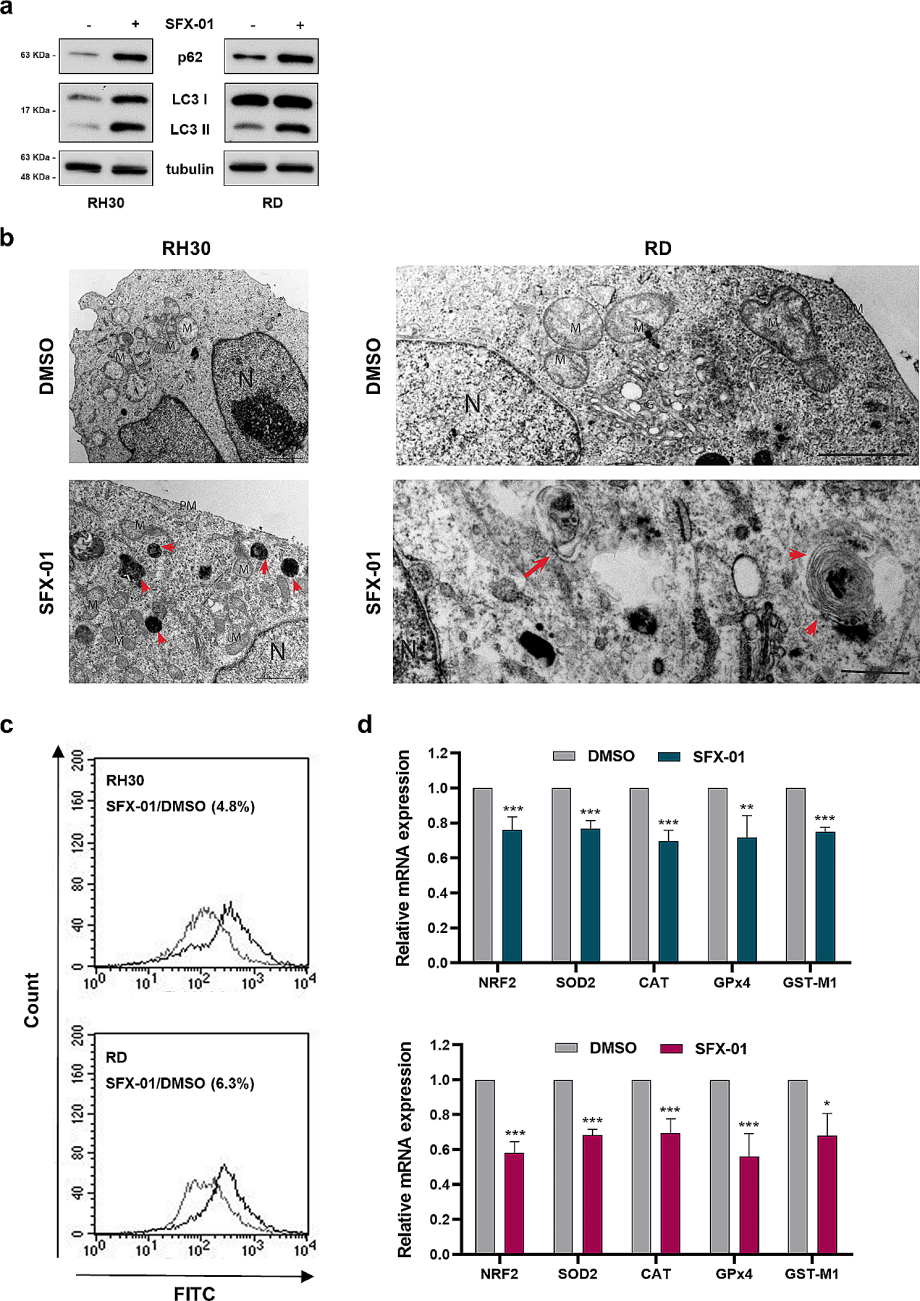
Effect of SFX-01 treatment on autophagy, ROS production and antioxidant gene expression. **(a)** Western blot analysis of autophagic markers p62 and LC3-I/II in RH30 and RD cells at 72 h post SFX-01 treatment. Tubulin expression was used as internal control. Western blots were cropped to improve the conciseness of the results. Representative blots of four independent experiments. The original Western blot images can be found in Supplementary File_uncropped. **(b)** TEM analysis to monitor autophagy in RH30 and RD cells exposed to SFX-01 or DMSO. Autophagosomes, characterised by double membrane, and lysosomal structures are indicated by the red arrows. Autolysosomes were not detected. N: nucleus; M: mitochondria; PM: plasma membrane. **(c)** Cytofluorimetric analysis of total reactive oxygen species produced by RH30 (upper panel) and RD (lower panel) cells 48 h post SFX-01 pre-incubation. **(d)** NRF2 transcript levels and specific downstream targets analysed by q-PCR assay in RH30 (upper panel), and RD (lower panel) cells treated with 10 µM SFX-01. Results are expressed as fold increase over DMSO treated cells, arbitrarily set at 1. GAPDH mRNA level was used as endogenous control. Data are expressed as mean values ± SD of four independent experiments, each performed in triplicate. Statistical analyses were performed by using Student’s t-test: *, *p* < 0.05; **, *p* < 0.01 and ***, *p* < 0.001 vs. DMSO

Since apoptosis and autophagy are two strictly regulated biological processes that play a key role in maintaining tissue homeostasis under both physiological and pathological conditions [44], we analysed the autophagic process. Levels of p62 and LC3-I/II, two canonical markers of autophagy, were assessed by Western blotting. As shown in Fig. 4a, we found a strong up-regulation of both p62 and LC3-II after SFX-01 treatment, this suggesting a suppression of autophagic processes at the late stage. Indeed, the increase of LC3-II indicates the autophagosome formation, but the concomitant accumulation of p62 reveals an impaired autophagic flux. The same trend of p62 and LC3-I/II modulation was also obtained in RH4 and JR1 cell lines, as reported in Supplementary Fig. 1d. In order to confirm this hypothesis, we performed TEM experiments for monitoring the number of autophagosomes and autolysosomes. In agreement with the Western blotting assays, in RMS cells treated for 72 h with SFX-01, we detected the presence of some autophagosomes, characterised by double membrane containing cytosol and/or morphologically intact organelles, and many lysosomal structures, but did not observe autolysosomes with organelles at various stages of degradation (Fig. 4b, Supplementary Fig. 4). Moreover, the images highlighted several mitochondria with altered morphology compared to DMSO-treated cells, which appeared without vacuoles and with normal mitochondria.

Since mitochondrial damage can be induced by oxidative stress, we assessed the ROS produced by RMS cells by using flow cytometry, and we demonstrated the increase of ROS levels in RH30 and RD cells exposed for 72 h to SFX-01 compared to DMSO controls (Fig. 4c). Since the transcription factor NRF2 plays a crucial role in the antioxidant defence of eukaryotic cells by inducing the transcription of antioxidant and cytoprotective genes, we evaluated the mRNA levels of NRF2 and specific NRF2 downstream detoxifying and antioxidant targets by q-PCR. We found that SFX-01 treatment is able to significantly reduce the expression levels of NRF2, SOD2, CAT, GPx4 and GST-M1 in both RH30 and RD cells (Fig. 4d).

All together, these data suggest that caspase-independent apoptosis induced by SFX-01 in RMS cells is triggered by the inhibition of autophagic process at the autophagosome-lysosome fusion step and the induction of oxidative stress by the alteration of NRF2-antioxidant signalling pathway.

### SFX-01 affects the migration and invasion of RMS cells and impairs the formation of 3D rhabdospheres

**Fig. 5:**
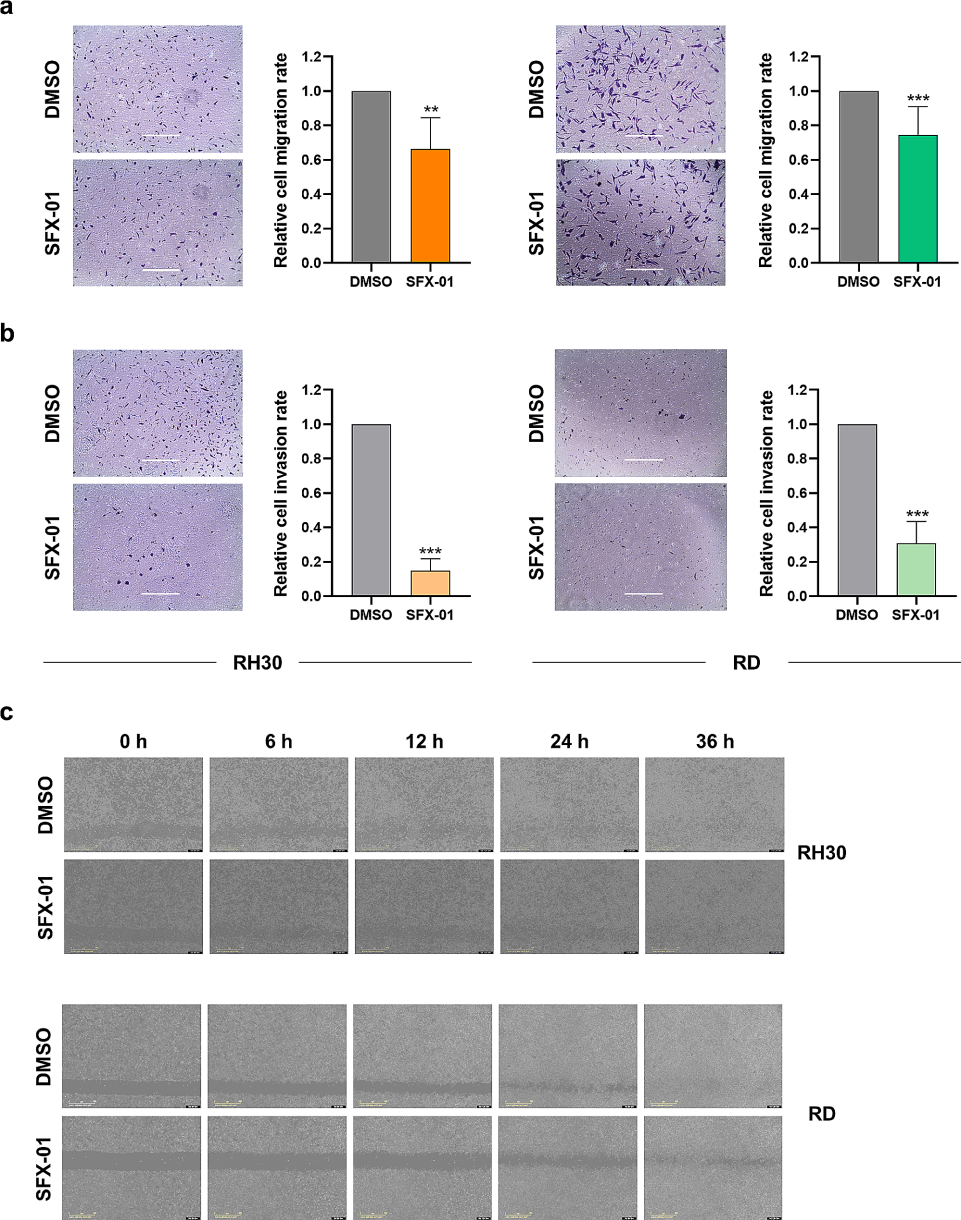
Migration and invasion ability in RH30 and RD cells after SFX-01 treatment. Trans-well migration **(a)** and invasion **(b)** assays in RH30 and RD cells exposed to SFX-01 or DMSO. Histograms showing the fold increase over DMSO treated cells, arbitrarily set at 1. Statistical analyses were performed by using Student’s t-test: **, *p* < 0.01 and ***, *p* < 0.001 vs. DMSO. Scale bar: 400 μm. **(c)** Representative imagines of Wound healing assays in RH30 and RD cells at different time points after SFX-01 incubation. Scale bar: 800 μm

To investigate whether SFX-01 exposure could also impair the metastatic progression of RMS cells, trans-well migration and invasion assay and wound-closure assay were performed. The results shown in Fig. 5 demonstrate that SFX-01 mediates a reduction of cell migration and a drastic suppression of cell invasion toward a chemoattractant. In particular, SFX-01 decreased the migration ability of RH30 cells of approximately 35% and RD cells of approximately 25% (Fig. 5a), whilst the invasion potential was inhibited of approximately 85% in RH30 cells and 70% in RD cells (Fig. 5b). Moreover, wound healing assay, performed photographing the same field immediately after the scratch (time 0 h) and again after 6, 12, 24 and 36 h following SFX-01 incubation, established that the specific treatment had a significant impact in the wound area closure only at 36 h after SFX-01 exposure (Fig. 5c) compared to DMSO treated cells (RH30, *p* = 0.003; RD, *p* = 0.03).

**Fig. 6:**
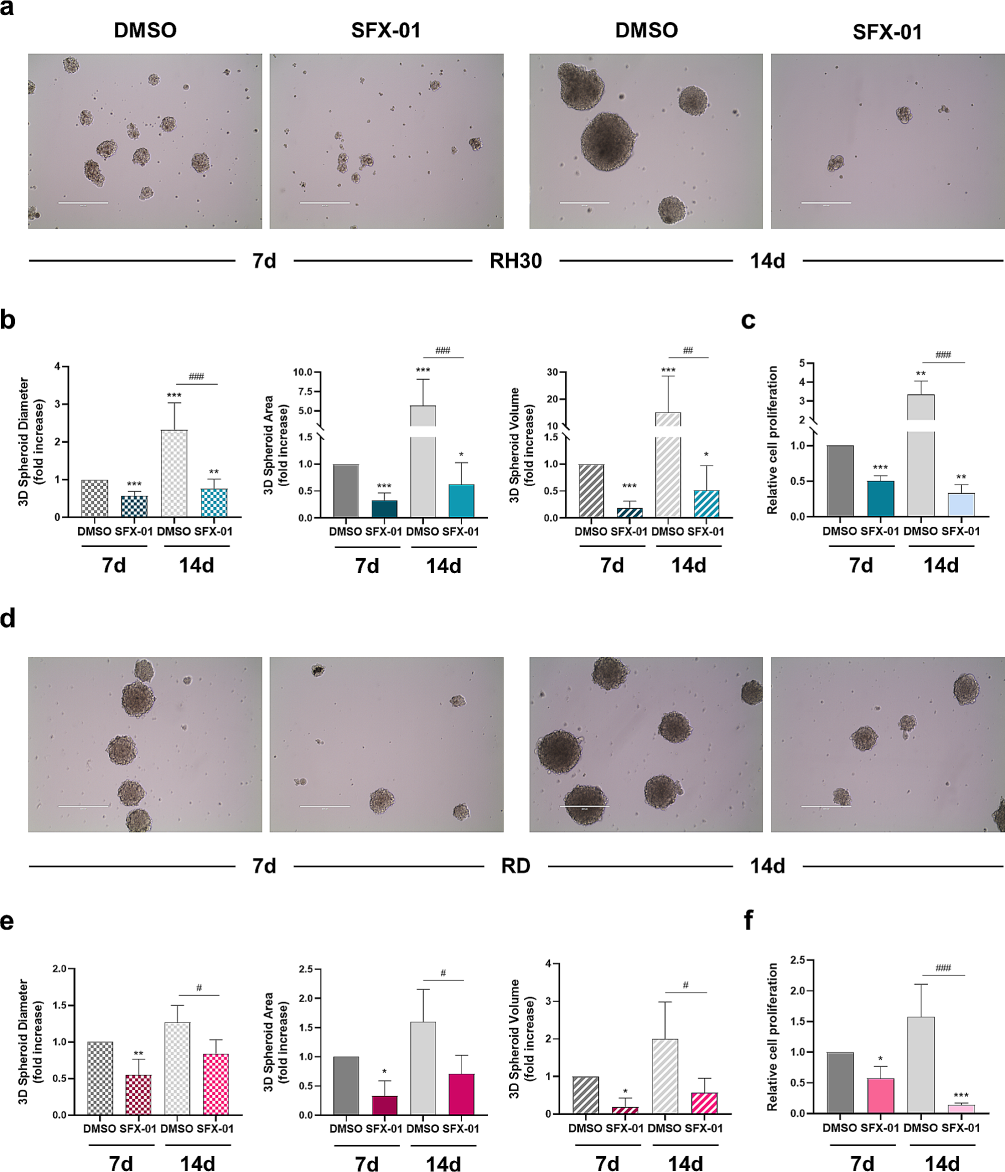
Assessment of RMS-derived spheroids after SFX-01 exposure. RH30 and RD cells were maintained in DMEM-F12 in ultra-low attachment plates and treated with 10 µM SFX-01 or DMSO. After 7 and 14 days, RH30 cells **(a)** and RD cells **(d)** were photographed for the evaluation of tumorsphere formation. Scale bar: 400 μm. Diameter, area and volume evaluation of RH30 **(b)** and RD **(e)** spheroids with AnaSP software. Histograms represent mean values ± SD of two independent experiments. Statistical analyses were performed by using Student’s t-test: *, *p* < 0.05, **, *p* < 0.01 and ***, *p* < 0.001 vs. DMSO at 7 days; ^#^, *p* < 0.05; ^##^, *p* < 0.01 and ^###^, *p* < 0.001 vs. DMSO at 14 days. **(c-f)** Trypan blue assay showing the proliferation of rhabdospheres at 7- and 14-days post SFX-01 exposure (10 µM) expressed as fold increase over DMSO treated cells (7 days), arbitrarily set at 1. Histograms represent mean values ± SD of three independent experiments. Statistical analyses were performed by using Student’s t-test: *, *p* < 0.05; **, *p* < 0.01 and ***, *p* < 0.001 vs. DMSO at 7 days; ^##^, *p* < 0.01 vs. DMSO at 14 days

Furthermore, to more accurately reproduce the complexity and heterogeneity of tumour cells as happens in the human body, we also used cells growing in 3D conditions [45]. So, the ability of SFX-01 to inhibit the 3D tumour spheroid formation was assessed in RH30 and RD cells maintained in ultra-low attachment plates. Cells photographed 7 and 14 days after SFX-01 pre-incubation suggested that the specific treatment counteracted the formation of 3D tumorspheres (Fig. 6a-d).

**Fig. 7:**
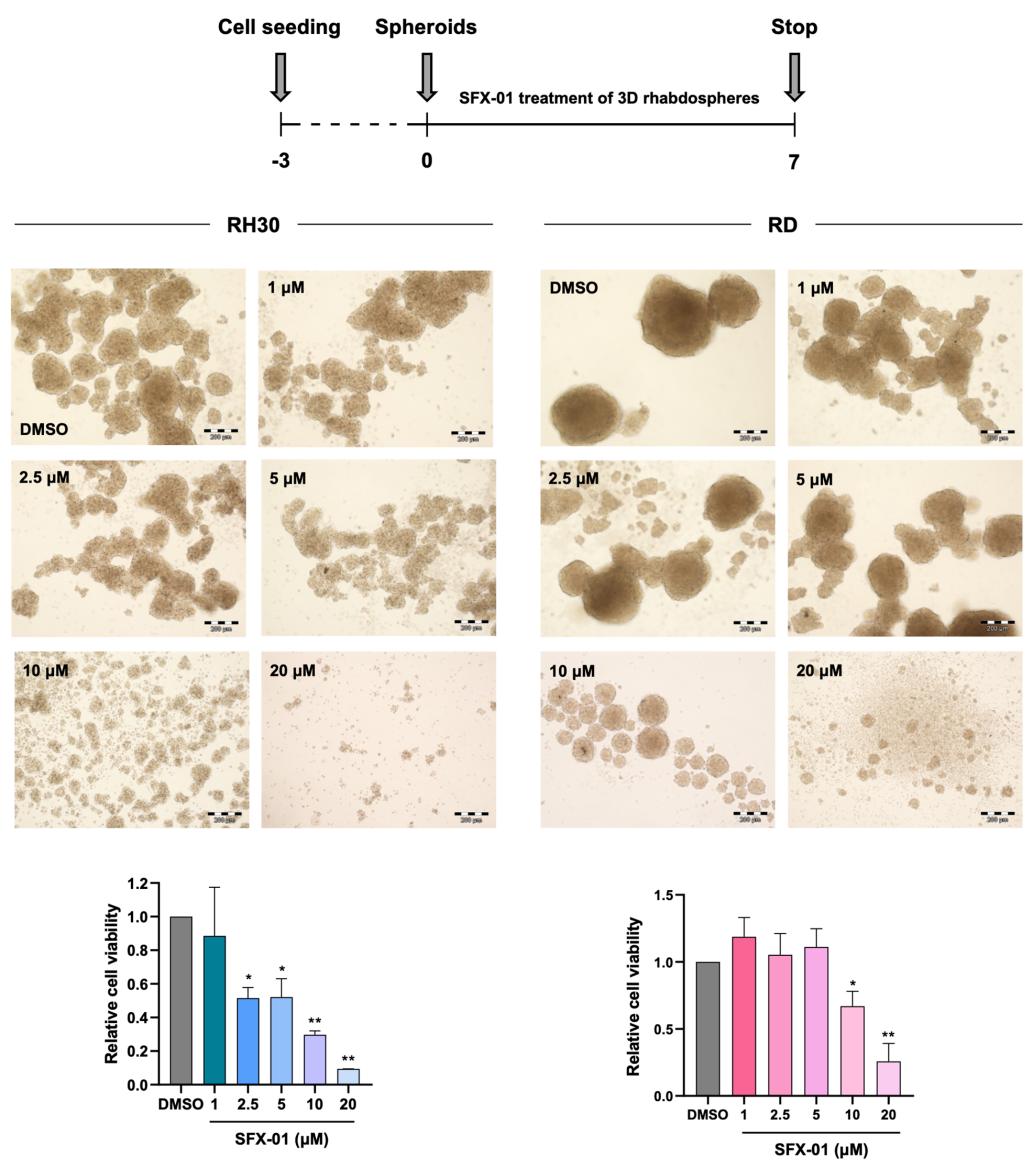
Evaluation of SFX-01 treatment on proliferation of RMS-derived spheroids. RH30 and RD cells maintained in DMEM-F12 in ultra-low attachment plates and treated with increasing concentration of SFX-01 or DMSO. After 7 days RH30 and RD cells were photographed (scale bar: 200 μm) and then were dissociated to assess cell vitality by trypan blue assay. Results were plotted as fold increase over DMSO treated cells, arbitrarily set at 1. Statistical analyses were performed by using Student’s t-test: *, *p* < 0.05 and **, *p* < 0.01 vs. DMSO

Indeed, we observed fewer 3D tumour spheroids and/or smaller rhabdospheres compared to the mocked controls, which, on the contrary, reached extremely large dimension after two weeks of culture. Specifically, the morphological analysis performed with the AnaSP software revealed that SFX-01 treatment led to a significative reduction of spheroid diameter, area and volume in both RH30 and RD cells after 7 days of culture (approximately 45%, 70% and 80%, Fig. 6b-e). At the end of the experiment, diameter, area and volume mean values of DMSO-treated spheroids showed a 2.3-, 6- and 15-fold increase in RH30 cells, and 1.3-, 1.5- and 2-fold increase in RD cells compared to negative control cells at day 7. On the contrary, 3D rhabdospheres exposed to SFX-01 exhibited drastically lower diameter, area and volume than DMSO-treated spheroids at 14 days, with a decrease of 65%, 90% and 95% in RH30 cells (*p* < 0.001, *p* < 0.001 and *p* = 0.003), and 40%, 56% and 70% in RD cells (*p* = 0.015, *p* = 0.02 and *p* = 0.03; Fig. 6b-e). Moreover, an evident disruption of the architectural structure of the spheroid population was observed in treated RH30 cells, the most aggressive subtype (Fig. 6a). In agreement with this result, the viability assay performed on dissociated spheroids by using Trypan blue dye exclusion test, demonstrated that SFX-01 exposure strongly reduced the survival of RMS-derived tumorspheres. As showed in Fig. 6c-f, the cell growth significantly decreased by 50% and 40% in RH30 and RD cells after 7 days of incubation and by 70% and 85%, respectively, at the end of the experiment. Moreover, in order to comprehensively assess the cytotoxic impact mediated by SFX-01 on tumour spheroid viability, we conducted experiments where spheres were allowed to form over a period of 3 days and subsequently by treating them with escalating concentrations of SFX-01 (1, 2.5, 5, 10 and 20 µM) or DMSO for 7 days (Fig. 7). Notably, a dose-response relationship was evident in both RH30 and RD cell lines, underscoring the potency of SFX-01 in significantly inhibiting RMS cell viability. Specifically, a reduction of approximately 50% was observed in RH30 cell number at 2.5 µM (*p* = 0.03) and 5 µM SFX-01 (*p* = 0.03), with a more pronounced effect at 10 µM (about 70%, *p* = 0.006) and 20 µM (nearly 90%, *p* = 0.002), whilst RD-derived spheroids exhibited significant disintegration at 10 µM (approximately 40%, *p* = 0.04) and 20 µM (about 80%, *p* = 0.004) of SFX-01 concentration.

Altogether these results suggest that SFX-01 may inhibit the dissemination of cancer cells throughout the body affecting the structural integrity and proliferation rate of circulating tumour masses.

### SFX-01-mediated inhibition of DNA damage repair pathway improves IR anticancer efficacy

**Fig. 8:**
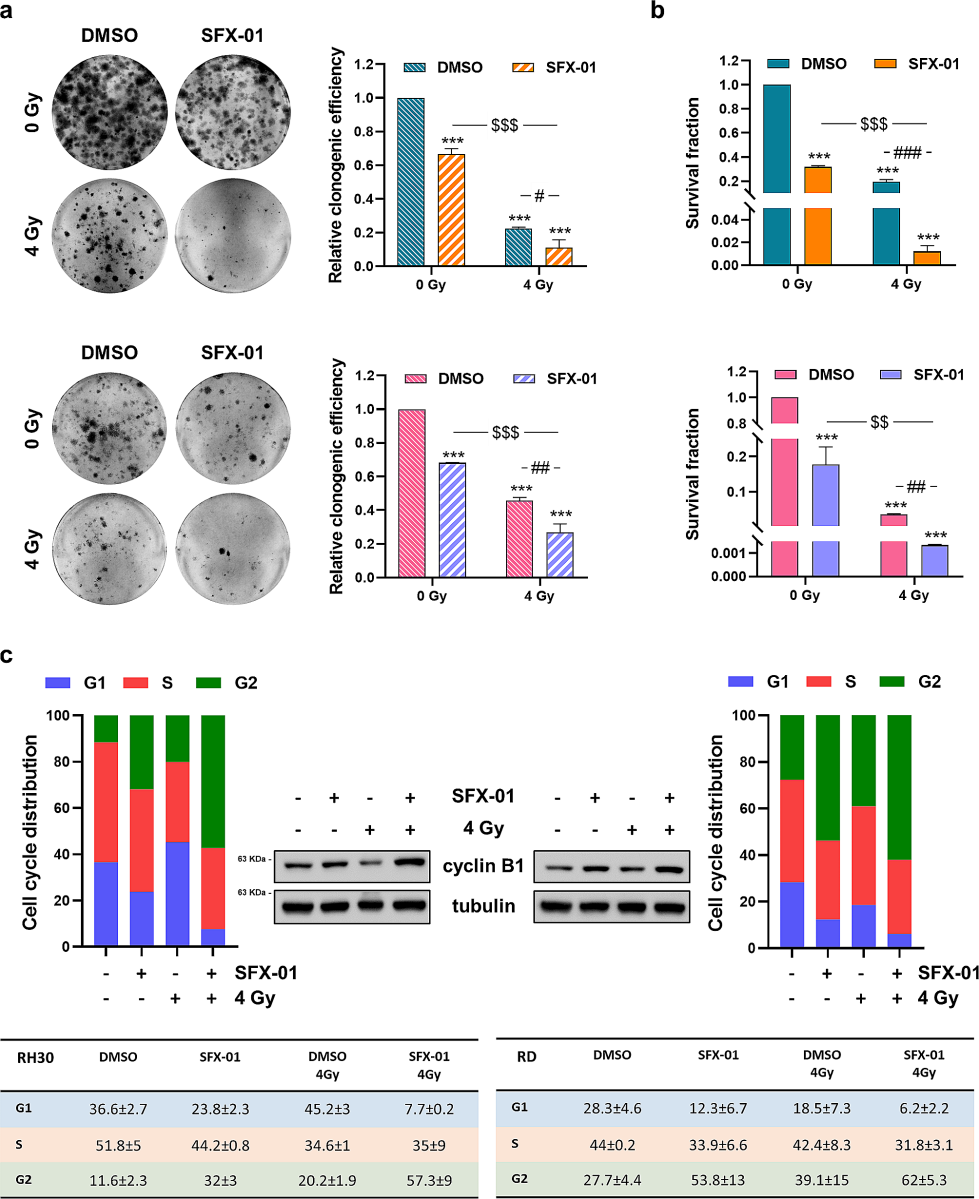
Evaluation of SFX-01/IR simultaneous treatment on clonogenic potential and proliferation of RMS cells. **(a)** Clonogenic ability of RH30 (upper panels) and RD (lower panels) cells treated or not with SFX-01 (10 µM) and exposed or not to IR (4 Gy). Representative pictures of colonies stained with crystal violet; histograms show the colony forming efficiency calculated by crystal violet absorbance from three independent experiments, each performed in triplicate. Each bar represents the means ± SD. Statistical analyses were performed by using two-way ANOVA: ***, *p* < 0.001 vs. DMSO/0 Gy; ^$$$^, *p* < 0.001 vs. SFX-01/0 Gy; ^#^, *p* < 0.05 and ^##^, *p* < 0.01 vs. DMSO/4 Gy. **(b)** MHDSA performed in RH30 (upper panel) and RD (lower panel) cells 11 days after the combined treatment by using Trypan blue dye exclusion test. Results were plotted as mean ± SD of two independent experiments. Statistical analyses were performed by using two-way ANOVA: ***, *p* < 0.001 vs. DMSO/0 Gy; ^$$^, *p* < 0.01 and ^$$$^, *p* < 0.001 vs. SFX-01/0 Gy; ^##^, *p* < 0.01 and ^###^, *p* < 0.001 vs. DMSO/4 Gy. **(c)** Histograms showing the cell cycle distribution of RH30 (left panel) and RD (right panels) cells pre-incubated with SFX-01 and exposed to IR. Mean values ± SD of each cell cycle phase of two independent experiments are reported in the tables. Western blot assay showing cyclin B1 up-regulation in RMS cells treated with SFX-01 and exposed to a single dose of 4 Gy. Tubulin expression was used as loading control. Representative blots of two independent experiments. Western blots were cropped to improve the conciseness of the results. The original Western blot images can be found in Supplementary File_uncropped

To assess whether SFX-01 treatment could sensitise RMS cells to IR, which are largely used for the management of paediatric patients with RMS tumours, RH30 and RD cells were pre-incubated with 10 µM SFX-01 for 24 h and subsequently exposed to a single dose of 4 Gy. To evaluate the effects induced by the combined treatment, colony formation ability of RMS cells was evaluated 11 days after plating the cells at very low-density and without adding fresh SFX-01. The assay highlighted that SFX-01 significantly reduced the number of colonies and improved the efficacy of IR in both the cell lines (Fig. 8a).

**Fig. 9:**
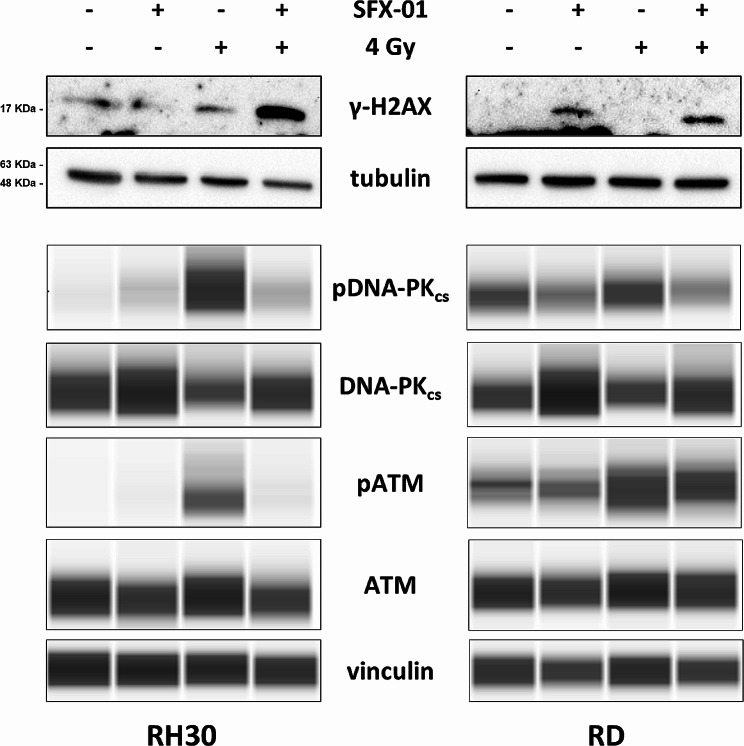
Analysis of specific markers of DNA damage and response pathway. γ-H2AX expression levels analysed by Western blot in RH30 and RD cells 48 h after SFX-01 treatment and 24 h post radiation exposure. Tubulin was used as loading control. Representative blots of three independent experiments. Western blots were cropped to improve the conciseness of the results. The original Western blot images can be found in Supplementary File_uncropped. Levels of phosphorylated and total form of DNA-PK_cs_ and ATM analysed by automated Western blot (Protein Simple WES Western technology) in RMS cells after the simultaneous treatment. Vinculin was used as internal control. Representative images of two independent experiments

Indeed, the clonogenic efficiency inhibition rate was approximately 35% in RH30 and RD cells treated with SFX-01, 80% and 55% in irradiated RH30 and RD cells and reached 90% and 75% in RH30 and RD cells exposed to the combined treatment. Since plating efficiency might decrease after irradiation, we also used the modified high-density survival assay. Specifically, 24 h after radiotherapy RMS cells were re-plated at high density in cell culture flasks for a prolonged period and the effect induced by the combined treatment was evaluated by counting the total number of cells. As showed in Fig. 8b the survival rate of RH30 and RD cells exposed to SFX-01, IR or both, was significantly lower compared to negative control cells and the synergistic effect previously detected with the clonogenic assay was even more evident. Indeed, SFX-01/IR combination led to a 0.01- and 0.001-fold decrease of cell survival compared to DMSO/0 Gy samples (*p* < 0.001) in RH30 and RD cells, respectively. To deeply understand the cytostatic activity exerted by SFX-01 pre-treatment combined with irradiation the cell cycle distribution of RH30 and RD cells was analysed by flow-cytometry. At 24 h post-radiation, we observed a statistically significant increase of cell percentage in G2 phase (RH30 *p* < 0.001; RD *p* = 0.004) and the corresponding increase of cyclin B1 protein levels (Fig. 8c). To investigate the specific mechanism underlying the radiosensitising properties of SFX-01 in RMS cells, the protein levels of several markers of DNA damage and response pathway were analysed by traditional or automated Western blot. The assays revealed the increase of γ-H2AX, a well-established marker of DNA Double Strand Breaks (DSBs) and the concomitant reduction of phosphorylated form of DNA-PK_cs_ and ATM, two proteins involved in the DNA damage repair, in RMS cells exposed to the simultaneous treatment compared to irradiated cells (Fig. 9). The reduced clonogenic ability and the γ-H2AX accumulation were also confirmed in RH4 and JR1 cells treated with SFX-01 + IR (Supplementary Fig. 5).

These data indicate that SFX-01 increases the effects of radiotherapy by drastically reducing the survival and proliferation of cancer cells and inhibiting the DNA repair.

### SFX-01 enhances the radiosensitivity of parental and clinically relevant radioresistant RMS-derived 3D spheroids and impairs tumour growth in mouse xenograft

**Fig. 10:**
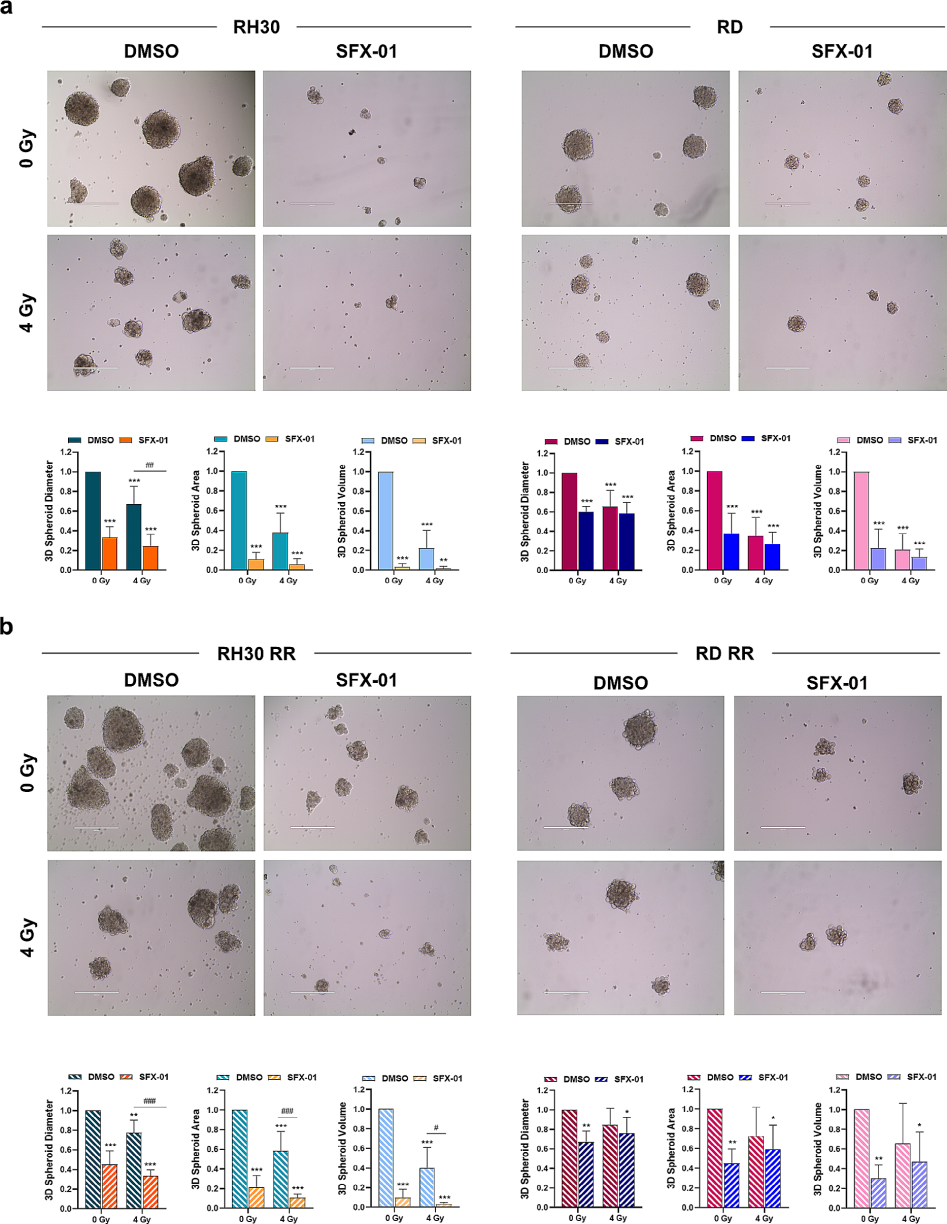
Spheroids formation in parental and clinically relevant radioresistant RMS cell lines. **(a)** Parental RH30 and RD cells and **(b)** radioresistant RH30 RR and RD RR cells maintained in DMEM-F12 in ultra-low attachment plates pre-treated with 10 µM SFX-01 or DMSO for 24 h and subsequently exposed or not to IR (4 Gy). After 14 days, cells were photographed for the evaluation of 3D spheroid formation. Scale bar: 400 μm. Lower panels showing the analysis of spheroid diameter, area and volume performed with AnaSP software on images acquired in two independent experiments. Bars represent the fold increase over DMSO/0 Gy sample, arbitrarily set at 1. Statistical analyses were performed by using two-way ANOVA: *, *p* < 0.05; **, *p* < 0.01 and ***, *p* < 0.001 vs. DMSO/ 0 Gy; ^#^, *p* < 0.05, ^##^, *p* < 0.01 and ^###^, *p* < 0.001 vs. DMSO/4 Gy

Since we demonstrated the ability of SFX-01 to affect the formation of 3D rhabdospheroids as single agent, we wondered if the treatment was also capable to increase the radiation sensitivity of RMS-derived tumorspheres. As highlighted in Fig. 10a rhabdospheres grown in ultra-low attachment plates, pre-incubated with 10 µM SFX-01 and irradiated with a single dose of 4 Gy, shown to be more sensitive to IR than single exposure alone, especially those derived from RH30 cells. Indeed, the automated analysis performed with AnaSP on morphological parameters of 3D spheroids revealed that IR alone significantly reduced diameter, area and volume by approximately 35%, 60% and 80% compared to mocked controls, whilst the combined treatment led to a decrease of approximately 75%, 94% and 98%. Moreover, the effect of SFX-01 treatment and/or IR exposure was also evaluated in clinically relevant radioresistant RMS cell lines (RH30 RR and RD RR), previously obtained in our laboratory [35]. As suggested by the results showed in Fig. 10b, SFX-01 also exerted its antitumour activity against spheroids derived from radioresistant RMS cells, significantly decreasing diameter (55% RH30 RR, 35% RD RR), area (80% RH30 RR, 55% RD RR) and volume (99% RH30 RR, 70% RD RR) of treated 3D rhabdospheres. More interestingly, SFX-01 acted synergistically with radiotherapy in RH30 RR cells. The representative images showed in Fig. 10b demonstrate that the radiation exposure led to a reduction in the 3D spheroid diameter, area and volume (approximately 25%, 40% and 60%) compared to the negative control spheroids (RH30 RR DMSO/0 Gy), but this decrease was significantly higher after the combined treatment (approximately 70%, 90% and 97%). As regards to RD RR cells, although we did not observe a synergistic effect, 3D spheroids treated with IR and pre-incubated with SFX-01 were smaller than those observed in both negative controls and irradiated samples, with a statistically significant reduction of morphological parameters, still suggesting a more positive outcome than radiation alone (Fig. 10b).

**Fig. 11:**
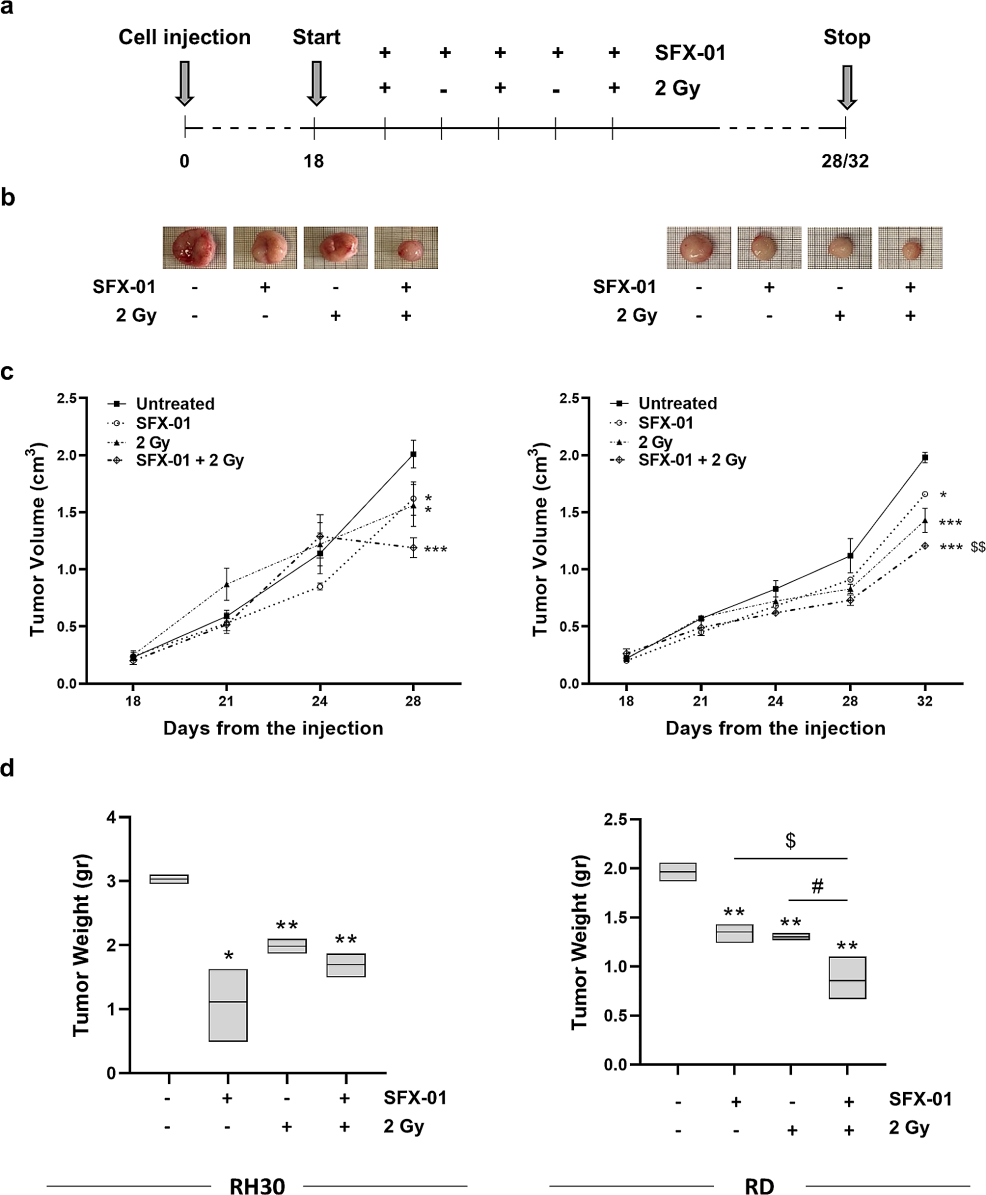
Effect of SFX-01/irradiation co-treatment on tumour growth in mouse xenograft. **(a)** Diagram illustrating the experiment procedure. Treatment started 18 days from cell injection, when tumours reached an initial volume of about 0.2 cm^3^. Mice received SFX-01 or vehicle by mouth once daily for five consecutive days and were irradiated, or not, every other day with a single dose of 2 Gy. **(b)** Images of RH30 and RD untreated, SFX-01, IR and SFX-01 + IR tumours explanted from mice post euthanasia at the end of the experiment. **(c)** Growth curves of tumour volumes from RH30 and RD xenografts collected from untreated, SFX-01-treated, irradiated (2 Gy), or co-treated (SFX-01 + 2 Gy) mice. Each point is the mean ± SD of three mice per group. Statistical analyses were performed by using two-way ANOVA: *, *p* < 0.05, **, *p* < 0.01 and ***, *p* < 0.001 vs. untreated; ^$$^, *p* < 0.01 vs. SFX-01. **(d)** Box plots showing tumour weights of RH30 and RD xenografts treated or not with SFX-01 and exposed or not to IR. Each box represents minimum to maximum and mean value of three mice per group. Statistical analyses were performed by using two-way ANOVA: *, *p* < 0.05 and **, *p* < 0.01 vs. untreated; ^$^, *p* < 0.05 vs. SFX-01; ^#^, *p* < 0.05 vs. 2 Gy

Finally, to assess the SFX-01 activity in vivo, xenografts of RH30 and RD cells were subcutaneously injected in nude mice. When the tumour volume reached 0.2 cm^3^ (day 18 from the injection), mice received SFX-01 (50 mg/kg) or vehicle (PBS) by mouth once daily for five consecutive days and were irradiated, or not, every other day with a single dose of 2 Gy (Fig. 11a). Tumour of xenografts from mice treated with SFX-01 significantly decreased compared to those of untreated mice, and the simultaneous SFX-01/IR treatment enhanced this reduction (Fig. 11b). SFX-01 treatment decreased the tumour volume by approximately 20% in both RH30 and RD xenografts compared to untreated mice (RH30 *p* = 0.05; RD *p* = 0.04), the radiation exposure led to a reduction of approximately 23% (*p* = 0.05) and 28% (*p* < 0.001) in mice receiving RH30 and RD cells respectively, and with combined treatment the decrease reached 40% (RH30 *p* < 0.001; RD *p* < 0.001) (Fig. 11c). Tumour weights exhibited the same trend. As shown in Fig. 11d, the mean weights of tumours harvested from RH30 xenografts were decreased by approximately 60% in SFX-01 treated mice (*p* = 0.02), by 35% in mice exposed to IR (*p* = 0.002) and about 45% in mice co-treated with SFX-01 and IR (*p* = 0.003) compared to untreated mice. Tumour weights collected from RD xenografts exposed to combined treatment significantly reduced compared to both untreated and single treated mice. In particular, SFX-01 or irradiation alone induced a decrease of about 20% (SFX-01 *p* = 0.009; IR *p* = 0.003) compared to negative controls, whilst the combined exposure induced a decrease of 50% (*p* = 0.008 vs. untreated; *p* = 0.02 vs. SFX-01; *p* = 0.02 vs. IR).

These results suggest that SFX-01 might represent a promising strategy to improve radiotherapy-mediated benefits and its efficacy against tumour progression.

### Discussion

Although oncology therapy has improved in the last decades, the conventional treatments do not kill all cancer cells, so tumour relapse and/or metastasis dissemination are still a major concern for all the patients, including children, and therefore alternative clinical protocols or innovative combined therapies are constantly under investigation. Plant-derived compounds have caught the interest of the scientific community and over the last few years SFN have been largely studied for their anticancer potential against different tumours. SFX-01, a complex of synthetic d, l-sulforaphane stabilised in alpha-cyclodextrin in phase I/II clinical trials for glioblastoma and breast cancer, is found to be safety and well tolerable, becoming a good candidate for being used in clinical settings. In this study, we analysed the biological and molecular effects of SFX-01 in two valuable models of RMS tumours, the most common soft tissue sarcoma in children. Our findings establish for the first time the cytostatic and cytotoxic effects mediated by SFX-01 in RMS cells and, its efficacy against 3D tumour spheroids both as a single agent and in combination with irradiation. We demonstrated that SFX-01 induced a marked decrease of cell proliferation in both RH30 and RD cell lines, ARMS and ERMS model respectively, which is correlated with a G2 cell cycle arrest supported by cyclin B1 up-regulation. The effect of SFX-01 on cell cycle distribution and progression, observed in our study, is in accordance with previous findings on osteosarcoma, prostate, colon and bladder cancer describing a G2/M phase arrest after treatment with SFN [27, 46–48]. Moreover, in the most aggressive ARMS subtype, we highlighted the modulation of specific cell cycle inhibitors, as the INK family member p16, p21 and p27 Cip/Kip proteins, in agreement with Bergantin et al. who reported p21 up-regulation at mRNA and protein levels only in ARMS models treated with 10 µM SFN for 12 h [31]. Remarkably, SFX-01 treatment restored p21 activation at transcriptional level despite the mutation and suppression of p53 that characterise RH30 cells [49]. Notably, the antiproliferative effect was not observed in non-malignant cells, demonstrating the specificity of SFX-01 for cancer cells. Indeed, trypan blue and Giemsa assays showed that SFX-01 does not impair either the HFM survival or their morphology.

Regarding cyclin D1 over-expression after SFX-01 treatment, our preliminary data suggest that cyclin D1 silencing improves the sensitisation of RH30 cells to SFX-01 as demonstrated by the significant decrease of viable cells, however it did not induce any change in RD cells. Over-expression of cyclin D1 at mRNA and protein levels after SFN exposure was also observed in lung cancer cells and its expression level was crucial for SFN-induced necrosis/apoptosis [50, 51]. These results indicate that cyclin D1 might be responsible for a primary cancer cell response to the drug, nevertheless our results clearly show the antitumour efficacy of SFX-01 in RH30 and RD cells. As previously reported [52], Cyclin D1 levels rapidly and persistently increased also in RMS cells by GLPG1790 treatment in comparison to mocked control cells with a perinuclear accumulation. Indeed, subcellular localization of Cyclin D1 outside the nucleus has been reported to correlate with a lower proliferative index in different cancer types, this suggesting that the restriction of Cyclin D1 to the perinuclear region may allow the suppression of cell cycle progression. Further experiments will be needed to elucidate the specific role of cyclin D1 in SFX-01-mediated effects observed in RMS.

Cancer cells have an elevated metabolism and higher levels of ROS than normal cells, but normally they maintain ROS at acceptable levels with an excellent antioxidant system including NRF2 transcription factor, the main regulator of intracellular redox homeostasis, and the antioxidant enzymes superoxide dismutase (SOD), catalase (CAT) and glutathione peroxidase (GPx) [53]. Thus, cancer cell killing by inducing oxidative stress represents a promising therapeutic strategy [54]. Indeed, muscle cells produce high level of ROS because of their contractile activity and metabolic demand, but abnormal ROS production and/or reduced antioxidant defences are involved in several pathological conditions [55, 56]. Since RMS is a malignant solid tumour that arises from mesenchymal progenitors that have lost the ability to differentiate into skeletal muscle cells, it should be more sensitive to therapeutic protocols causing oxidative stress due to high endogenous ROS levels [57, 58]. In this study, we found that SFX-01 induces caspase-independent cell death mediated by inhibition of autophagy and induction of oxidative stress. Indeed, we detected (i) PARP but not caspase 3 activation as well as the concomitant LC3-II and p62 over-expression by Western blot, (ii) autophagosomes but not autolysosomes as well as damaged mitochondria by TEM, (iii) increased levels of total ROS by FACS analysis and finally (iv) reduced levels of NRF2 transcript and NRF2-downstream targets by q-PCR.

On the other hand, radiotherapy, one of the gold standards for RMS treatment, mainly kills cancer cells by increasing oxidative stress and intracellular ROS levels, which represent the main induction mechanism of DNA DSBs, so we hypothesised that SFX-01 could enhance the efficacy of IR and reduce radioresistance mechanisms. The results demonstrated that the combination treatment is a promising therapeutic strategy to increase antitumour effects of radiotherapy in RMS. RH30 and RD cells, pre-incubated with SFX-01 and then exposed to radiation, significantly reduced the ability of the cells to form colonies and survive for a long period. Since radiosensitivity correlates with DSB number at 24 h after irradiation [59] and accumulation of γ-H2AX, a well-established marker of DNA DSBs, which was detected in SFX-01/IR treated samples compared to only irradiated cells, our results suggest that the drug treatment impairs the DNA damage repair signalling pathway. Phosphorylated protein levels of DNA-PK_cs_ and ATM were analysed to confirm the lower activation of non-homologous end joining (NHEJ) and homologous recombination (HR) pathways respectively, which represent the main molecular mechanisms orchestrating the DNA damage response (DDR) in eukaryotic cells. An important finding of the present study is the ability of SFX-01 to impair the formation and proliferation of 3D tumour spheroids and to enhance the sensitivity of both parental and radioresistant cell-derived rhabdospheres to radiotherapy. Three-dimensional spheroids more accurately recapitulate some important in vivo features of solid tumours such as internal structure, cellular heterogeneity, cell polarization, extracellular matrix deposition, cell-to-cell and cell-to-extracellular matrix interactions, hypoxia and drug penetration, allowing a better prediction of response or resistance to therapy than conventional 2D cell cultures, and filling the gap between in vitro studies and animal models [60–63]. Our data on morphological parameters of 3D spheroids suggest that SFX-01 can prevent rhabdosphere establishment as highlighted by the modulation of shape and structural integrity of spheroids, which exhibited a corresponding reduction in the proliferation rate.

Moreover, 3D tumour spheroids correlate with cancer stem cells (CSCs), a sub-population of tumour cells with self-renewal capacity responsible for cancer development, progression, resistance to therapies, and tumour relapse. Several studies established CSC targeting by SFN in other tumour types [64] and SFX-01 itself reduces CSCs in breast cancer and glioblastoma [32, 34], So, our preliminary result on 3D spheroids represent a promising starting point for a better understanding of SFX-01 induced effects on RMS-derived CSCs. We also showed that SFX-01 treatment significantly reduces migration and invasiveness of RH30 and RD cells, highlighting the possible impact of this compound in preventing drug resistance, metastasis, and tumour relapse. Finally, our experiments gave a primarily evidence that SFX-01 impaired tumour growth in both ARMS and ERMS mouse xenografts, as suggested by the reduced tumour volumes and weights obtained from SFX-01 treated mice compared to negative control animals. Furthermore, SFX-01 radiosensitised ERMS cells in this in vivo model, as indicated by the ability of IR to significantly counteract tumour growth more efficiently in SFX-01 pre-treated mice. ARMS xenografts showed the same trend, but the comparison between combined treatment and single treatment was not statistically significative. While observed progressive disease response with slight growth delay in the mouse treated groups may suggest limited efficacy at this stage, it’s important to consider that our study represents an initial exploration of the antitumor effects of SFX-01 in RMS by using preclinical in vivo models that provide valuable insights but may not always fully recapitulate the complexity of human disease. Anyway, despite the observed growth delay, our study also provides mechanistic insights into the antitumor effects of SFX-01, including its impact on cellular pathways and potential synergistic interactions with radiotherapy. The observed in vivo results could also be due to the limited number of mice per treatment group used for the in vivo experiments, which represent as a starting point to establish proof-of-concept and to provide preliminary data for subsequent studies with larger sample sizes. Several published articles in the field of preclinical oncology also employ similar sample sizes of three mice per treatment group in order to adhere to the principles of the 3Rs (Replacement, Reduction, Refinement) for ethical animal research. Anyway, increasing the number of animal tests in future studies will allow to strengthen statistical power and reliability of the findings as well as to better define the therapeutic window of the optimal drug dosage and the potential long-term side effects, which could offer valuable insights before proceeding to clinical trials in RMS patients in order to ensure the safety and efficacy of SFX-01, both as a monotherapy and in combination with radiotherapy. Moreover, to further explore our results and gain insight into the exact molecular mechanisms potentially differently activated by which SFX-01 exerts its antitumour effects in ARMS and ERMS cells, we will perform RNAseq analysis on the tumour masses harvested from treated mice, which will be presented in a future manuscript.

## Conclusions

In conclusion, the present study demonstrated for the first time the antitumour activity of SFX-01 in two preclinical models of ARMS and ERMS tumours. In particular, SFX-01 treatment significantly reduced cell proliferation, migration and invasion of RMS cells, and enhanced the radiotherapy efficacy by affecting the DNA damage repair machinery. Moreover, the strong cytotoxic effects, induced by the combined treatment, drastically inhibited the formation of RMS-derived 3D spheroids of both parental and clinically relevant radioresistant RMS cells. Finally, we have indication of the ability of SFX-01 to reduce the in vivo growth of tumour masses in RMS mouse xenografts, both as a single agent and combined with irradiation. Altogether, the obtained results suggest SFX-01/IR co-treatment as a novel approach with potential therapeutic benefits, mainly in the clinical management of patients with aggressive RMS disease.

### Electronic supplementary material

Below is the link to the electronic supplementary material.

Supplementary Material 1

Supplementary Material 2

## Data Availability

The datasets used and/or analysed during the current study are available from the corresponding author on reasonable request.

## Abbreviations

RMS: Rhabdomyosarcoma
ROS: Reactive oxygen species
IR: Ionising radiation
ARMS: Alveolar rhabdomyosarcoma
ERMS: Embryonal rhabdomyosarcoma
EFS: Event-free survival
SFN: SulforaphaneE
DMEM-HG: High Glucose Dulbecco’s Modified Eagle Medium
FBS: Foetal bovine serum
HFM: Human foetal myoblast
RR: Relevant radioresistant
DMSO: Dimethyl sulfoxide
H: Hours
Min: Minutes
RT: Room temperature
PI: Propidium iodide
siRNA: Small interfering RNA
q-PCR: Real Time PCR
SDS-PAGE: Sodium dodecyl sulphate-polyacrylamide gel
PVDF: Polyvinylidene fluoride
BSA: Bovine serum albumin
PBS-T: Phosphate buffered saline with Tween 20
3D: Three dimensional
TEM: Transmission electron microscopy
MHDSA: Modified high density survival assay
EC: European Community
DSB: Double Strand Breaks
SOD: Superoxide dismutase
CAT: Catalase
GPx: Glutathione peroxidase
NHEJ: Non-homologous end joining
HR: Homologous recombination
DDR: DNA damage response
CSCs: Cancer stem cells
