# Supplementary material for: Antitumour effects of SFX-01 molecule in combination with ionizing radiation in preclinical and in vivo models of rhabdomyosarcoma
Source: BMC Cancer. 2024 Jul 8;24:814. doi: 10.1186/s12885-024-12536-8 (PMC11229215; doi:10.1186/s12885-024-12536-8)
Supplement: Supplementary file 1 — Supplementary Material 1 [file 12885_2024_12536_MOESM1_ESM.pdf]

Supplementary Files

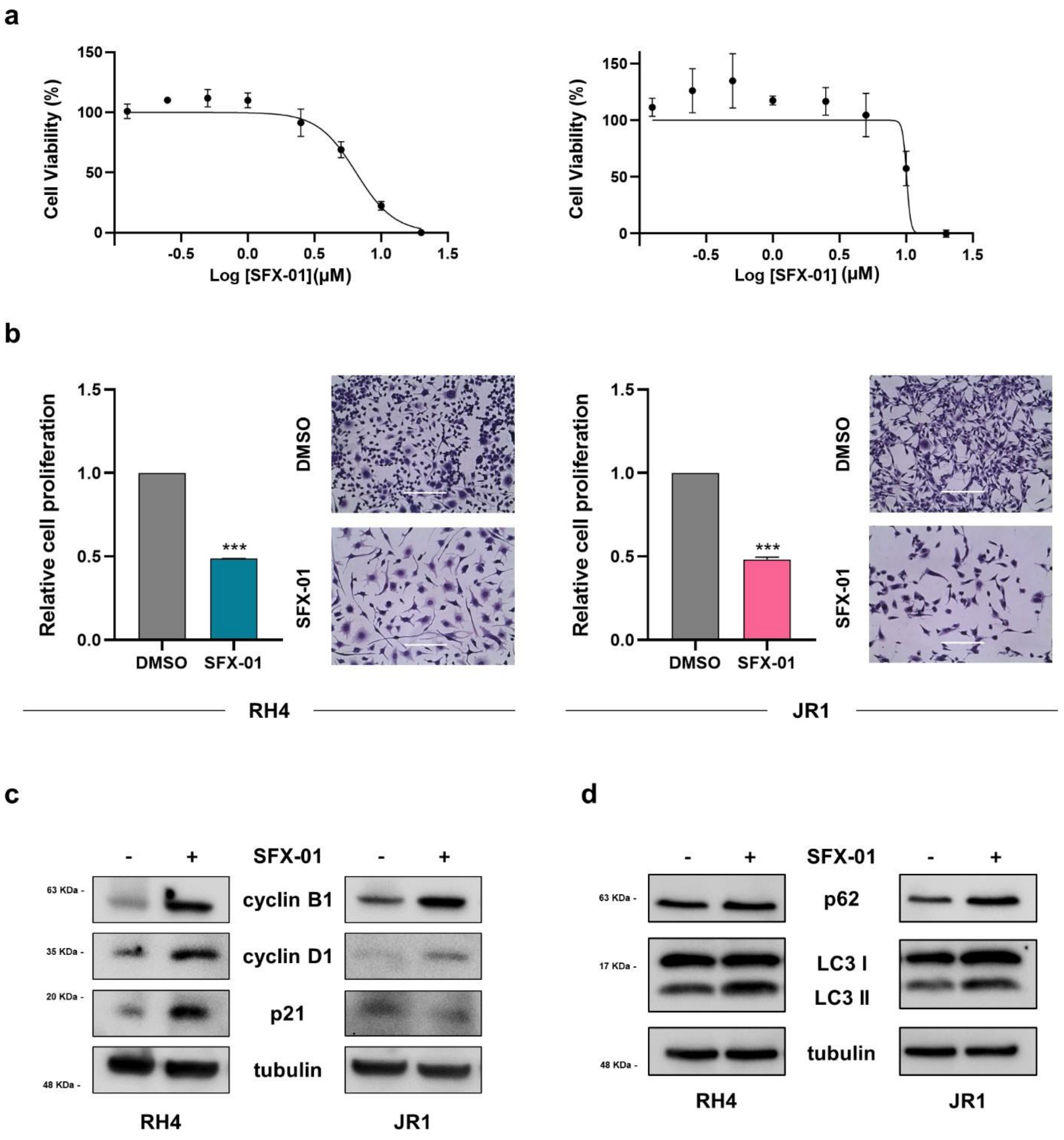

Supplementary Figure 1. SFX-01-induced effects on proliferation, morphology and autophagic processes in RH4 and JR1 cells. (a) *MTT assay performed on RH4 and JR1 cells treated for 72 h with increasing concentrations of SFX-01. Each point is the mean ± SD of two independent experiments each performed in sextuplicate.* ***(b)*** *Trypan blue assay showing RH4 and JR1 cell proliferation at 72 h post SFX-01 exposure (7.5 and 10 μM) expressed as fold increase over DMSO treated cells, arbitrarily set at 1. Histograms represent mean values ± SD of two independent experiments. Statistical analyses were performed by using Student's t-test: ***, p<0.001 vs. DMSO. Images showing the morphological assessment by Giemsa staining. Scale bar: 200 μm.* **(c)** *Cyclin B1, cyclin D1 and p21 protein expression* analysed with

Western blot in RH4 and JR1 cells treated with SFX-01 for 72 h. Tubulin expression was used as internal control. *Western blots were cropped to improve the conciseness of the results. The original Western blot images can be found in Supplementary File_uncropped.* ***(d)*** *Western blot analysis of autophagic markers p62 and LC3-I/II in RH4 and JR1 cells at 72 h post SFX-01 treatment. Tubulin expression was used as loading control. Western blots were cropped to improve the conciseness of the results. The original Western blot images can be found in Supplementary File_uncropped.*

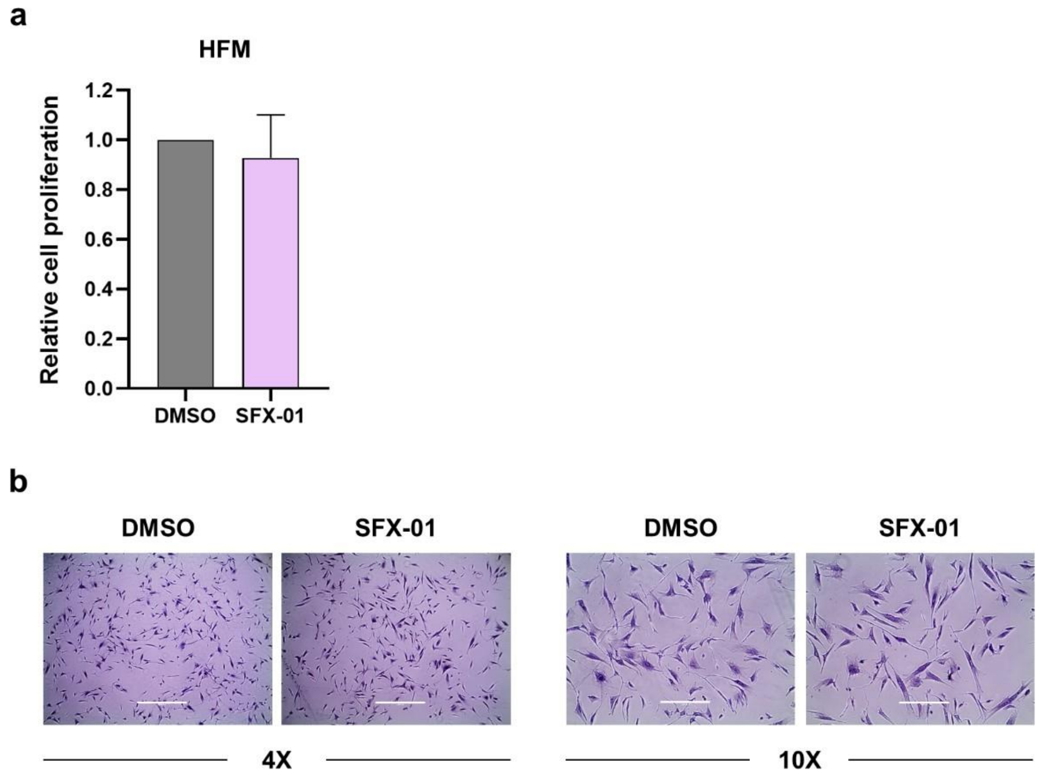

Supplementary Figure 2. Proliferation and morphology evaluation in HFM exposed to SFX-01. (a) *Trypan blue assay showing HFM (human foetal myoblast) proliferation at 72 h post SFX-01 treatment expressed as fold increase over DMSO treated cells, arbitrarily set at 1. Histograms represent mean values ± SD of two independent experiments.* ***(b)*** *Images showing the morphological assessment by Giemsa staining at 4x and 10x magnification. Scale bar: 1000 μm and 400 μm.*

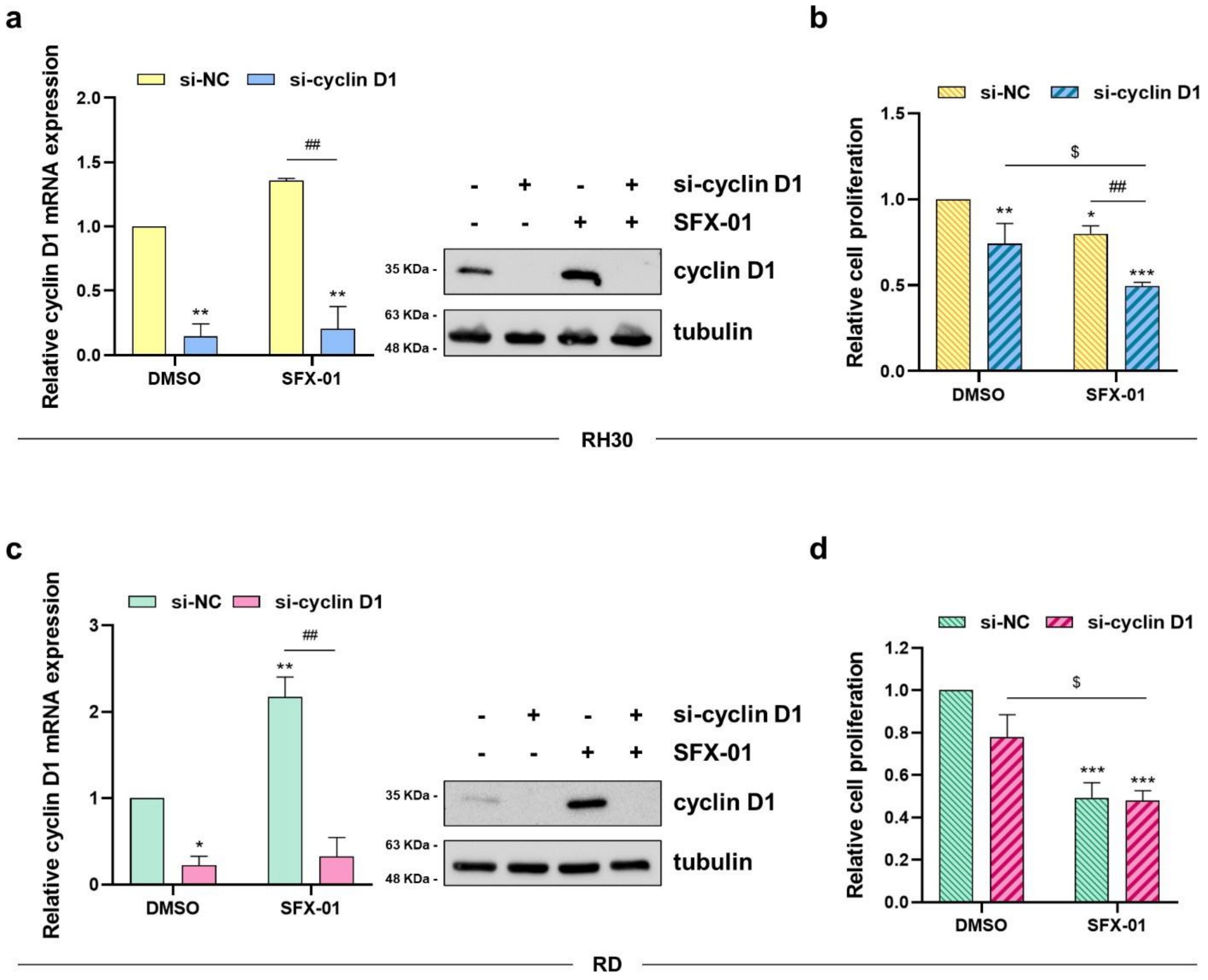

Supplementary Figure 3. Effect of cyclin D1 silencing and SFX-01 treatment on RMS cell proliferation. *Transcript levels (left panels) and protein expression (right panels) of cyclin D1 in RH30* ***(a)*** *and RD* ***(c)*** *cells transfected with si-cyclin D1 or si-NC for 72 h and treated with SFX-01 or DMSO. q-PCR analysis was expressed as fold increase over si-NC/DMSO arbitrarily set at 1, and GAPDH was used as endogenous control. Bars represent mean values ± SD of two independent experiments, each performed in triplicate. Statistical analyses were performed by using two-way ANOVA: *, p<0.05 and **, p<0.01 vs. si-NC/DMSO;* $^{\#\#}$*, p<0.01 vs. si-NC/SFX-01. For Western blotting assay, tubulin was used as loading control. Western blots were cropped to improve the conciseness of the results. The original Western blot images can be found in Supplementary File_uncropped. Relative cell proliferation in RH30* ***(b)*** *and RD* ***(d)*** *cells after cyclin D1 knocking down and SFX-01 treatment analysed by trypan blue dye exclusion test. Results are mean values ± SD of two independent experiments expressed as fold increase over si-NC/DMSO, arbitrarily set at 1. Statistical analyses were performed by using two-way ANOVA: *, p<0.05, **, p<0.01 and ***, p<0.001 vs. si-NC/DMSO;* $^{\$}$*, p<0.05 vs. si-cyclin D1/DMSO;* $^{\#\#}$*, p<0.01 vs. si-NC/SFX-01.*

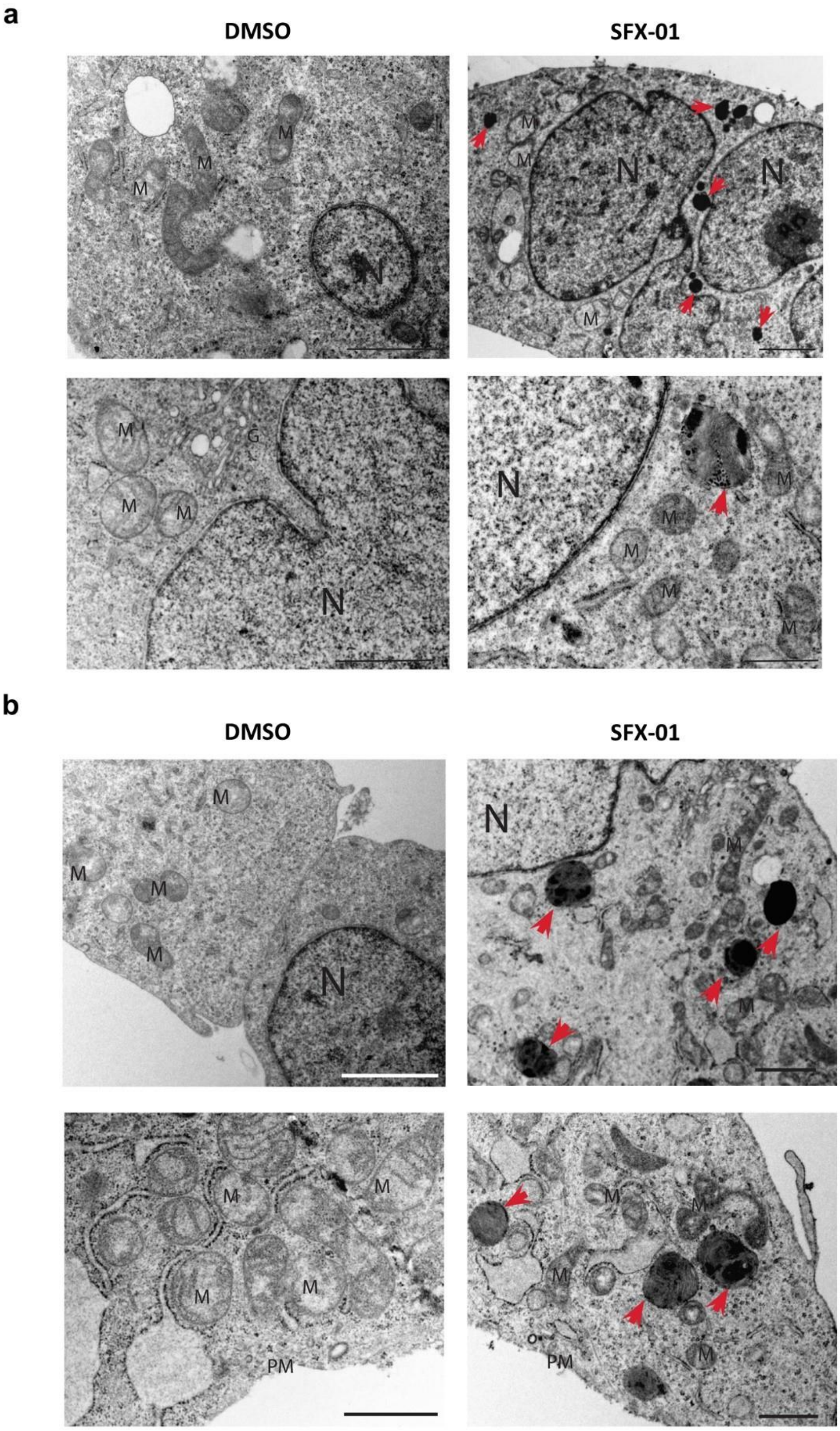

Supplementary Figure 4. TEM analysis to monitoring autophagy in RH30 and RD cells exposed to SFX-01. *Different images of **(a)** RH30 and **(b)** RD cells treated with SFX-01 or DMSO for 72 h. Autophagosomes, characterised by double membrane, and lysosomal structures are indicated by the red arrows. Autolysosomes were not detected. N: nucleus; M: mitochondria; PM: plasma membrane.*

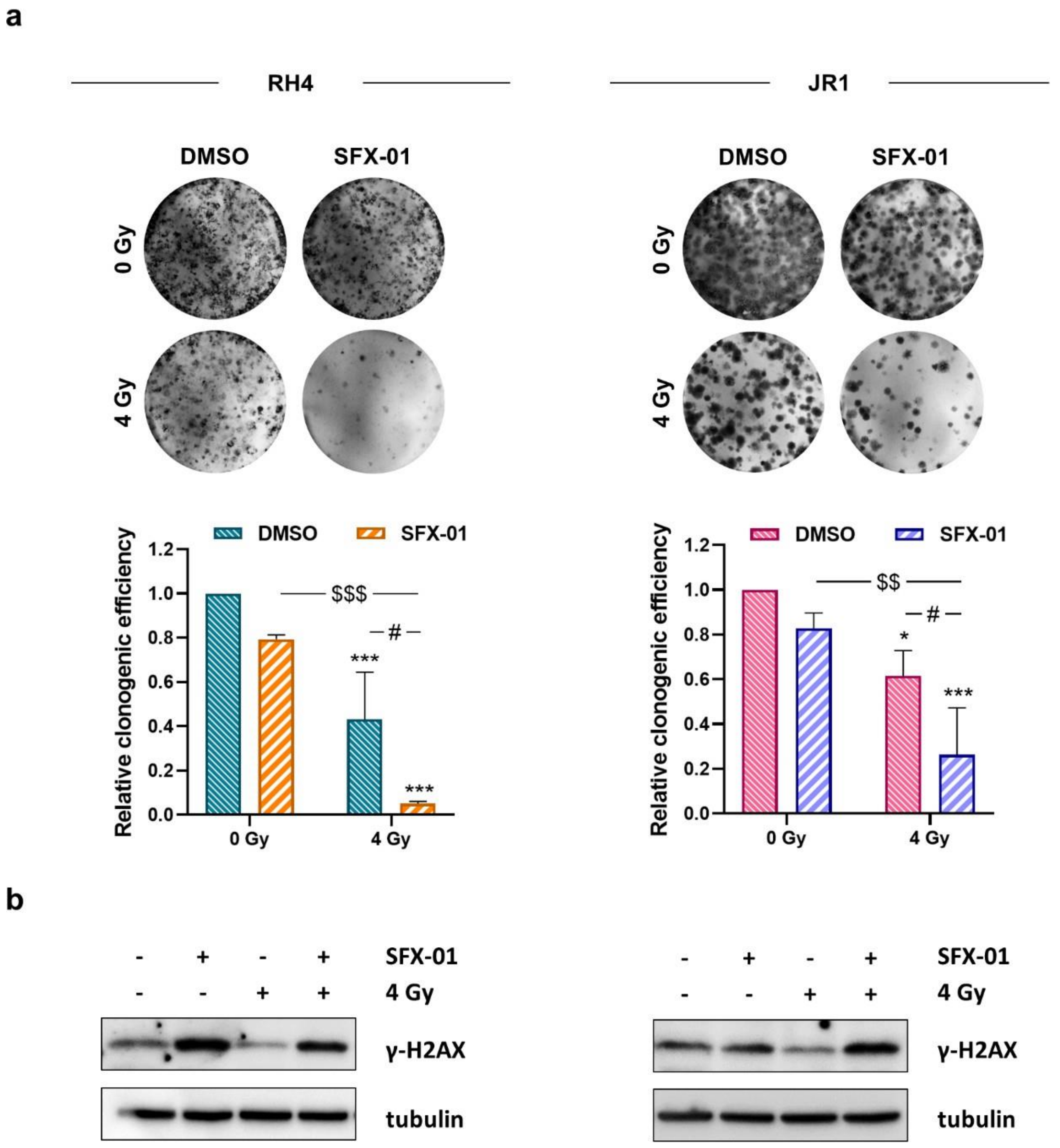

Supplementary Figure 5. Evaluation of clonogenic potential and DNA damage after SFX-01/IR combined treatment in RH4 and JR1 cells. (a) *Clonogenic ability of RH4 (left panels) and JR1 (right panels) cells treated or not with SFX-01/IR. Representative pictures of colonies stained with crystal violet; histograms show the colony forming efficiency calculated by crystal violet absorbance from two independent experiments, each performed in triplicate. Each bar represents the means ± SD. Statistical analyses were performed by using two-way ANOVA: ***, p<0.001 and **, p<0.01 vs. DMSO/0 Gy;* $^{\$\$\$}$*, p<0.001 vs. SFX-01/0 Gy;* $^{\#\#}$*, p<0.01 and* $^{\#\#\#}$*, p<0.001 vs. DMSO/4 Gy.* ***(b)*** *Western blot analysis of γ-H2AX protein levels in RH4 and JR1 cells 48 h after SFX-01 treatment and 24 h post radiation exposure. Tubulin was used as loading control. Western blots were cropped to improve the conciseness of the results. The original Western blot images can be found in Supplementary File_uncropped.*
