# Supplementary material for: Antitumour effects of SFX-01 molecule in combination with ionizing radiation in preclinical and in vivo models of rhabdomyosarcoma
Source: BMC Cancer. 2024 Jul 8;24:814. doi: 10.1186/s12885-024-12536-8 (PMC11229215; doi:10.1186/s12885-024-12536-8)
Supplement: Supplementary file 2 — Supplementary Material 2 [file 12885_2024_12536_MOESM2_ESM.pdf]

Supplementary File_uncropped

Ponceau S staining and merge (hybridization + standard) of uncropped blots showed in the final manuscript. The standard used in the experiments are HyperPAGE Prestained Protein Marker (BIOLINE) or Opti-Protein XL Marker (Applied Biological Materials).

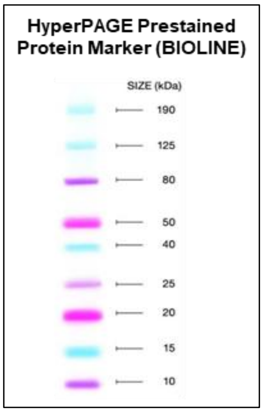

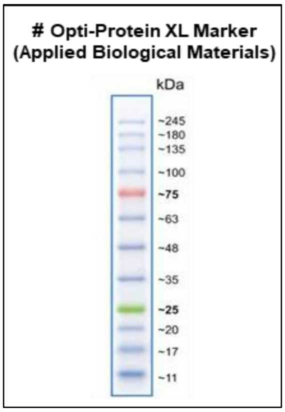

Figure 2b

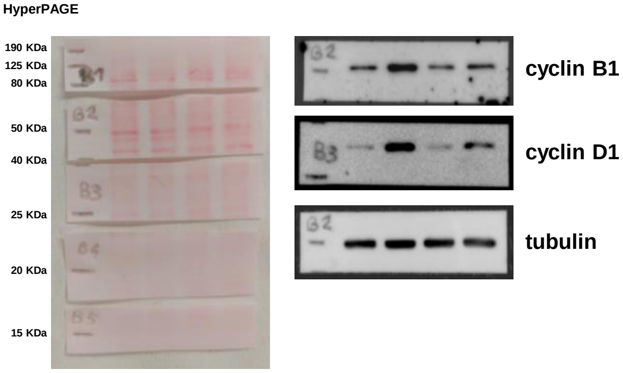

Figure 2c

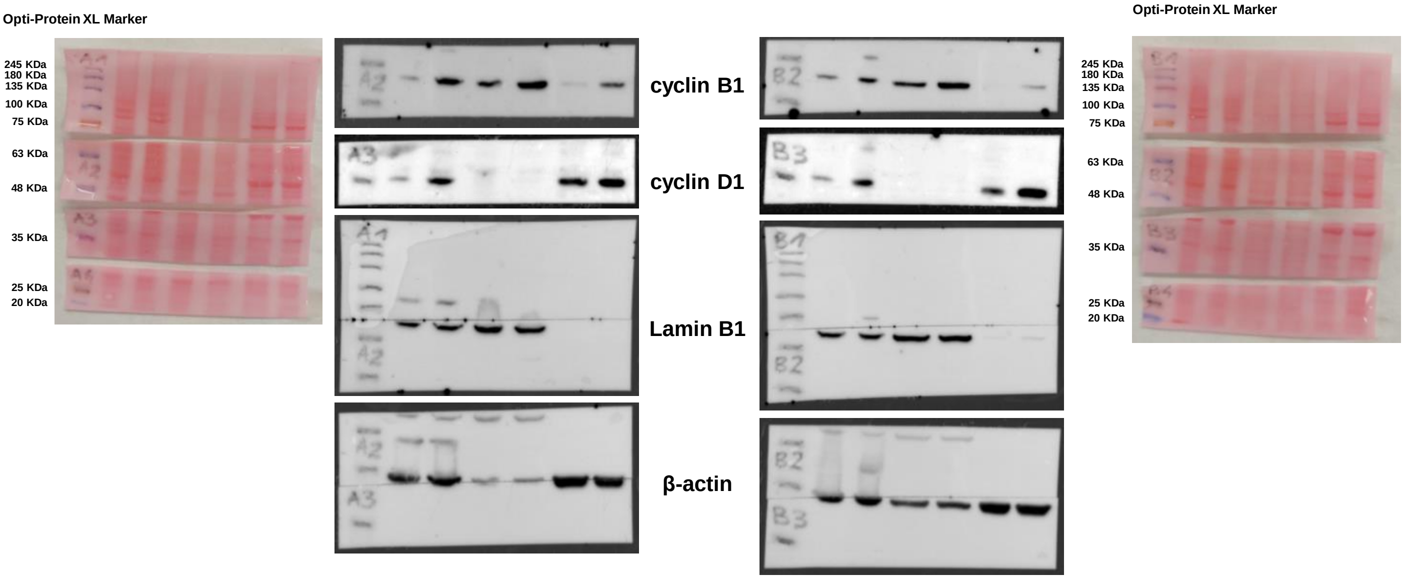

RH30 cells: lanes 3-4-5-6 are samples of the figure

RD cells: lanes 3-4-5-6 are samples of the figure

Figure 2d

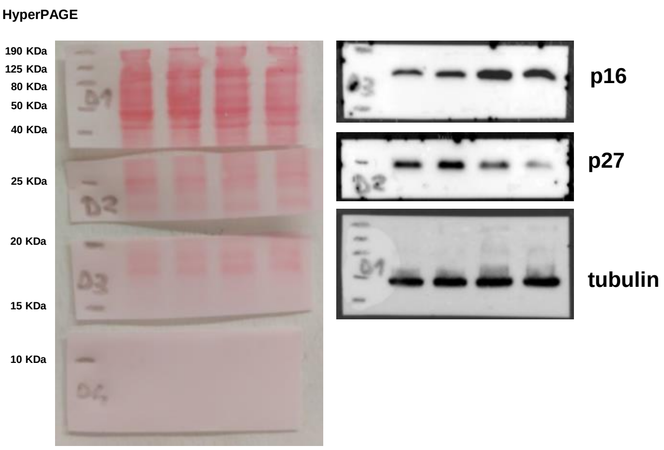

RH30 cells: lanes 1-2 are samples of the figure

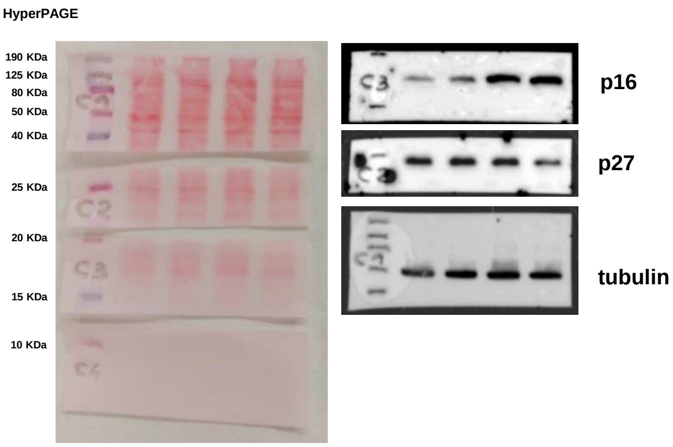

RD cells: lanes 3-4 are samples of the figure

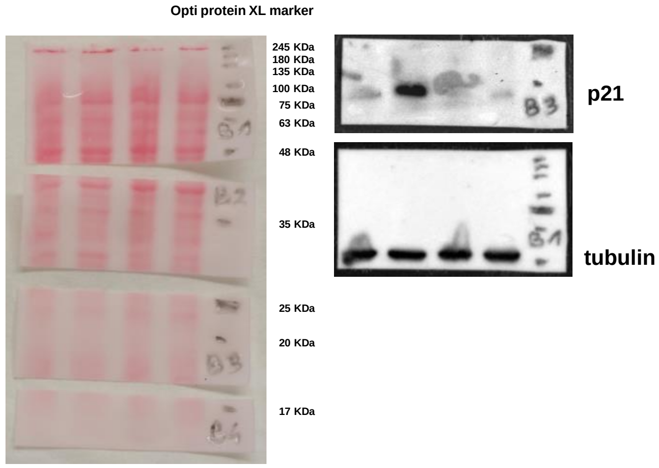

Figure 3b

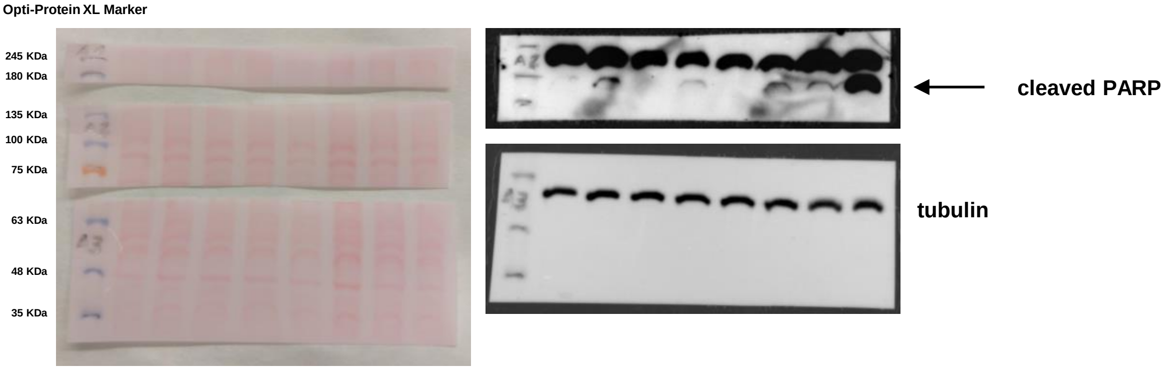

Lanes 1-2 (RH30 cells) 5-6 (RD cells) are samples of the figure

Figure 4a

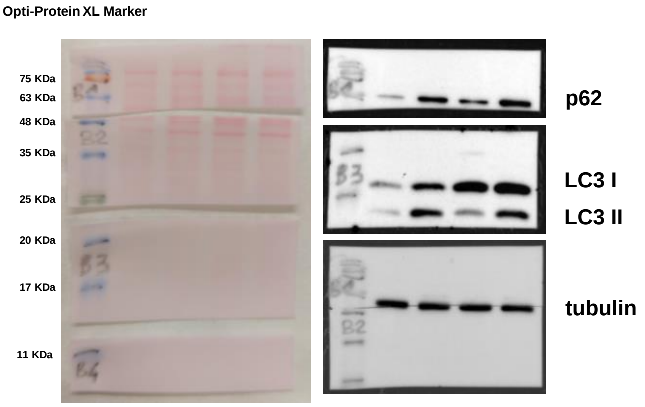

Figure 8c

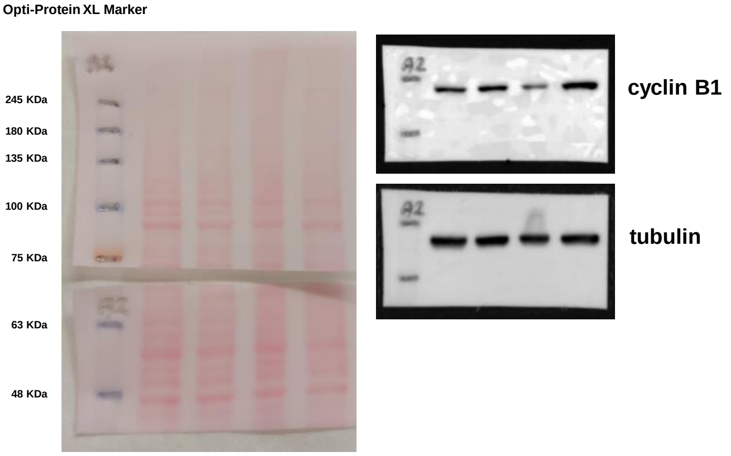

RH30 cells

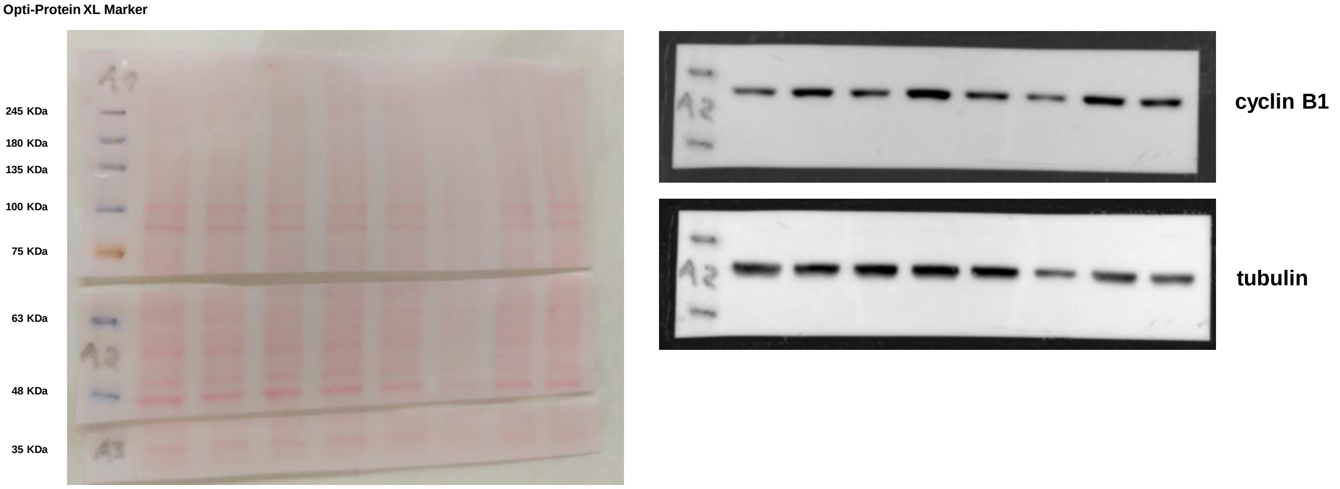

RD cells: lanes 1-2-3-4 are samples of the figure

Figure 9

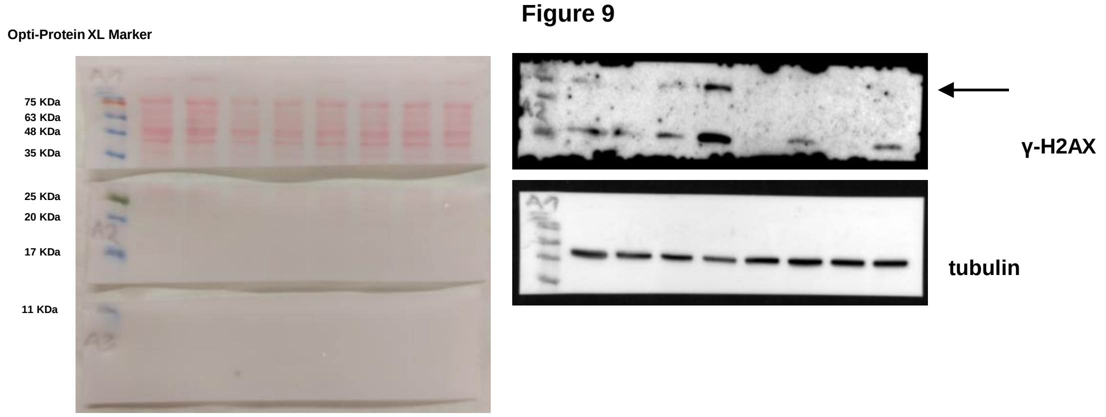

Supplementary Figure 1c

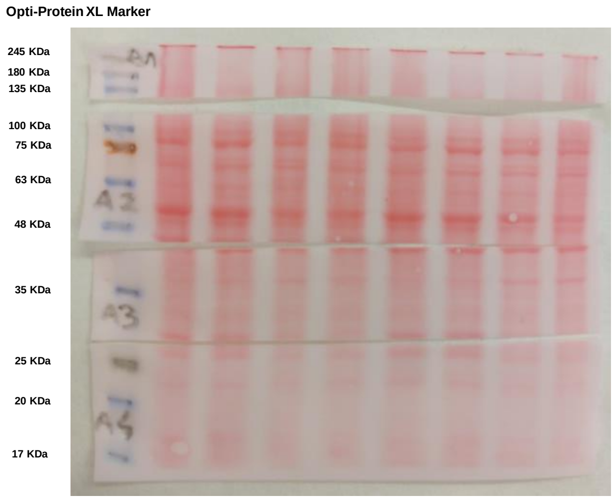

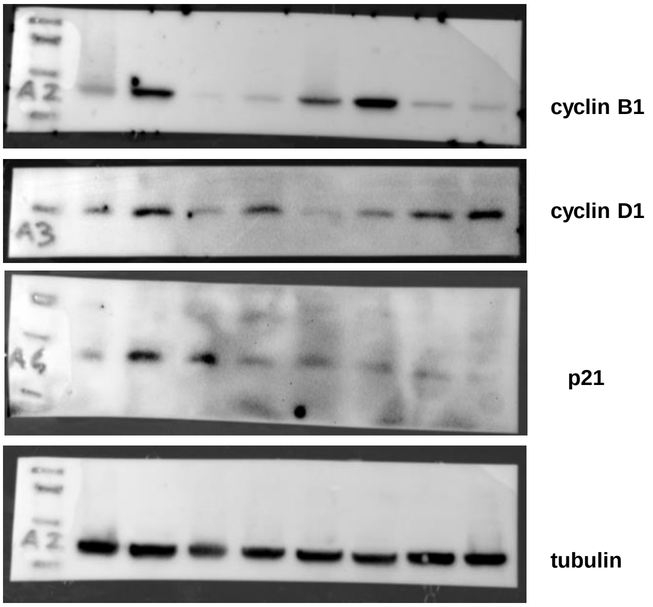

RH4 cells: lanes 1-2 are samples of the figure

JR1 cells: lanes 5-6 are samples of the figure

Supplementary Figure 1d

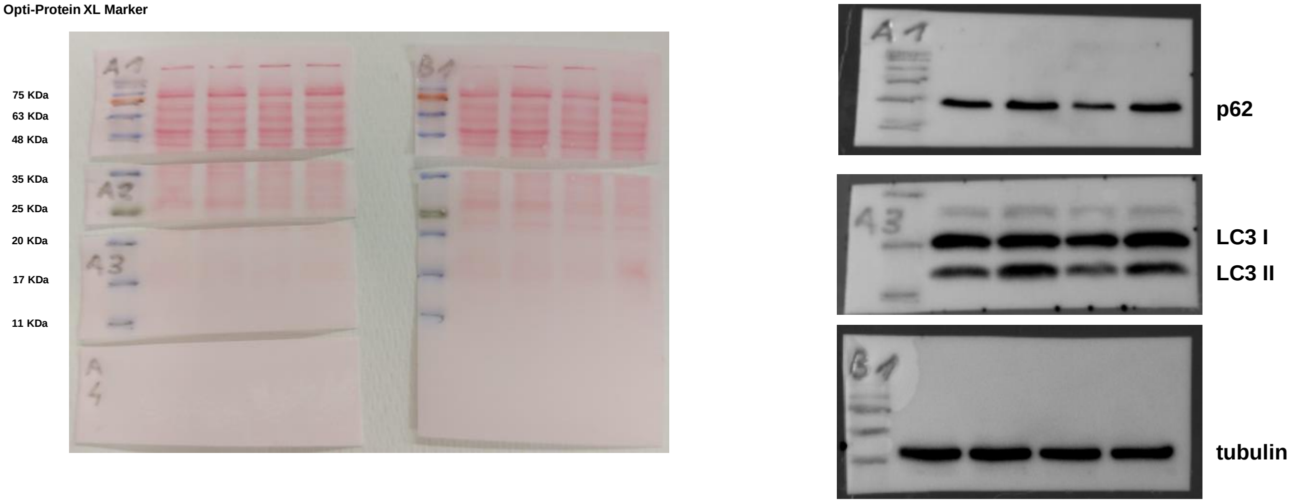

Protein lysates from the same experiment were run in different lanes of the same gel (A-B) and were probed with the specific antibodies (p62/LC3 I/II) or tubulin.
RH4 cells: lanes 1-2 (A and B) are samples of the figure; JR1 cells: lanes 3-4 (A and B) are samples of the figure

Supplementary Figure 3a

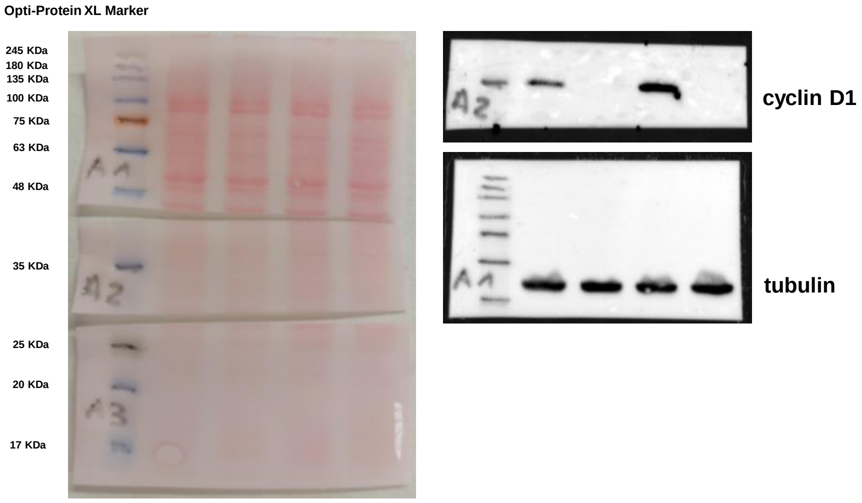

Supplementary Figure 3c

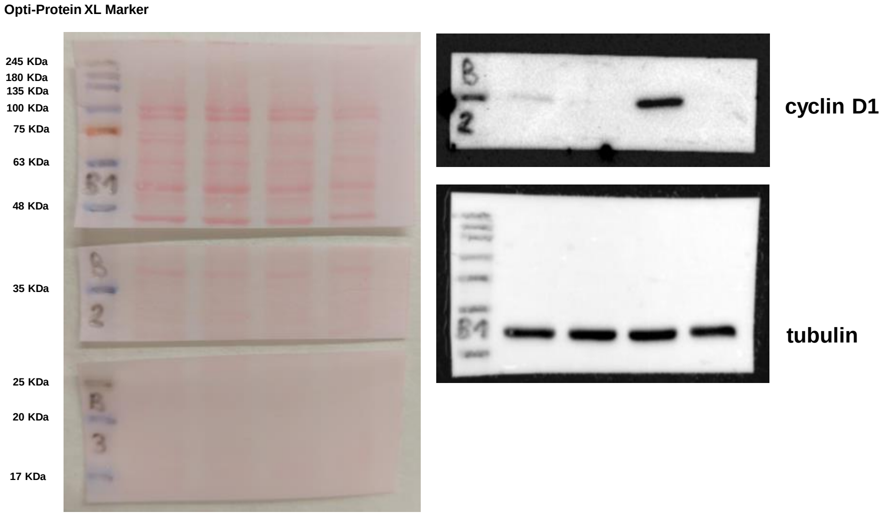

Supplementary Figure 5

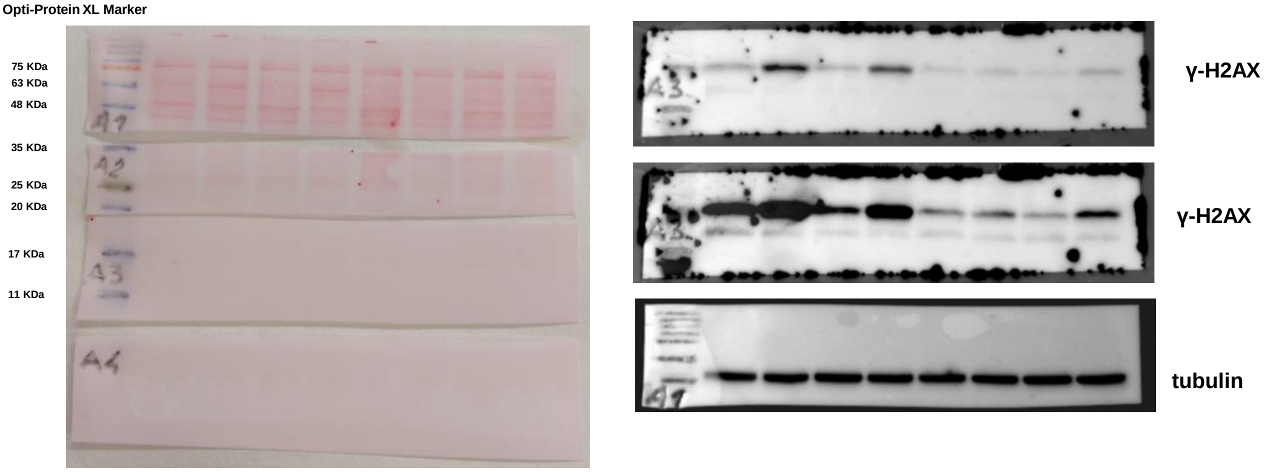

RH4 cells: lanes 1-2-3-4 are samples of the figure

JR1 cells: lanes 5-6-7-8 are samples of the figure
